# The roles of wing color pattern and geography in the evolution of Neotropical Preponini butterflies

**DOI:** 10.1002/ece3.6816

**Published:** 2020-10-03

**Authors:** Elena Ortiz‐Acevedo, Juan Pablo Gomez, Marianne Espeland, Emmanuel F. A. Toussaint, Keith R. Willmott

**Affiliations:** ^1^ Departamento de Química y Biología Universidad del Norte Barranquilla Colombia; ^2^ Florida Museum of Natural History University of Florida Gainesville FL USA; ^3^ Zoological Research Museum Alexander Koenig Bonn Germany; ^4^ Natural History Museum of Geneva Geneva Switzerland

**Keywords:** butterfly diversification, Lepidoptera phylogenetics, Neotropical biogeography, wing color pattern evolution

## Abstract

Diversification rates and evolutionary trajectories are known to be influenced by phenotypic traits and the geographic history of the landscapes that organisms inhabit. One of the most conspicuous traits in butterflies is their wing color pattern, which has been shown to be important in speciation. The evolution of many taxa in the Neotropics has also been influenced by major geological events. Using a dated, species‐level molecular phylogenetic hypothesis for Preponini, a colorful Neotropical butterfly tribe, we evaluated whether diversification rates were constant or varied through time, and how they were influenced by color pattern evolution and biogeographical events. We found that Preponini originated approximately 28 million years ago and that diversification has increased through time consistent with major periods of Andean uplift. Even though some clades show evolutionarily rapid transitions in coloration, contrary to our expectations, these shifts were not correlated with shifts in diversification. Involvement in mimicry with other butterfly groups might explain the rapid changes in dorsal color patterns in this tribe, but such changes have not increased species diversification in this group. However, we found evidence for an influence of major Miocene and Pliocene geological events on the tribe's evolution. Preponini apparently originated within South America, and range evolution has since been dynamic, congruent with Andean geologic activity, closure of the Panama Isthmus, and Miocene climate variability.

## Graphical abstract

We explore the potential role of phenotypic traits and major biogeographical events in explaining diversification dynamics and current distribution patterns of one of the most spectacularly colored butterfly groups in the Neotropics. We found that even though forewing color evolved fast in particular clades, these changes did not have a major influence on diversification dynamics. In contrast, we found that the Andean uplift was responsible for an increase in speciation rate and being responsible for current distribution patterns.

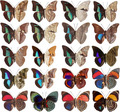

## 1 INTRODUCTION

Some phenotypic traits may serve as key innovations that facilitate rapid diversification, influencing the evolutionary origins and trajectories of lineages (Mayhew, 2007; Nicholson et al., 2014; Schluter, 2000). One of the most visually spectacular phenotypic traits is the bright color patterns in several insect groups, and a number of studies have focused on the evolution of these color patterns and the possible roles they might have played in the evolution of insects (Berthier, 2005; Jiggins et al., 2001; Kemp, 2007; Mallet & Gilbert, 1995). In butterflies, for example, wing color pattern is a trait that has attracted the attention of naturalists since Darwin's and Wallace's observations in the 19th century (Darwin, 1874, 1880; Wallace, 1877, 1889), and it has proven to be key in intra‐ and interspecific interactions and speciation processes in butterflies (e.g., Lycaenidae; Fordyce et al., 2002; *Heliconius*; Jiggins et al., 2006; *Heliconius*; Mavarez et al., 2006).

As an alternative to trait‐dependent diversification, geographic events can also promote diversification and range evolution. Understanding the biogeography of diverse Neotropical clades is therefore also key to obtain insights about the origins of extant biodiversity. There have been many changes in Neotropical landscape's configuration that have influenced the distribution and evolutionary trajectories of organisms (Antonelli et al., 2018; Hoorn et al., 2010). For example, the Andean uplift that started around 30 Ma has been one of the key drivers of speciation and range evolution in the Neotropical biota (Smith et al., 2014). This uplift created major rearrangements of internal wetlands that provided novel habitats and were important barriers to dispersal (Chazot et al., 2019; Rahbek et al., 2019). The formation of the Panama Isthmus was another major biogeographical event that promoted the great biotic American interchange shaping current Neotropical biodiversity (Bacon et al., 2015).

Phylogenetic methods now make possible the investigation of the relative contributions of phenotypic traits and biogeographical events on the evolutionary history of different groups of organisms (Pinto‐Sanchez et al., 2012; Weir et al., 2009). Combining knowledge of phylogenetic relationships, the timing of diversification and trait and distribution data permits testing of competing hypotheses about the origin and evolutions of diverse biotas (Mullen et al., 2011), and identifies traits that might have had a crucial role in the evolutionary history of organisms (Losos et al., 1997).

Butterflies are an excellent model system for the study of color evolution and the influence of geographic events on diversification. Color pattern alone can be used to distinguish most of the 18,000 described butterfly species (Nijhout, 1991). The most studied functions of color include mimicry, predator avoidance, mate recognition, and sexual selection, and these functions are thought to have influenced diversification (Chazot et al., 2014; Jiggins, 2008; Kemp, 2007; Obara & Majerus, 2000). Similarly, novel habitats and barriers to dispersal created by Neotropical landscape rearrangement have influenced the evolution of a number of butterfly groups (Chazot et al., 2016, 2019; Condamine et al., 2012; De‐Silva et al., 2016, 2017; Toussaint et al., 2019). The increasing availability of comprehensive dated phylogenetic hypotheses for butterflies (Chazot et al., 2020; Espeland et al., 2018) allows a more rigorous study of how different, and potentially conflicting, functions of color have generated trait and species distributions and diversity (Finkbeiner et al., 2014). Phylogenies also enable tests of how shifts in color pattern, in concert with changes in geographic range and habitat, have influenced speciation and diversification (Chazot et al., 2014; Jiggins et al., 2006).

**Figure 1:**
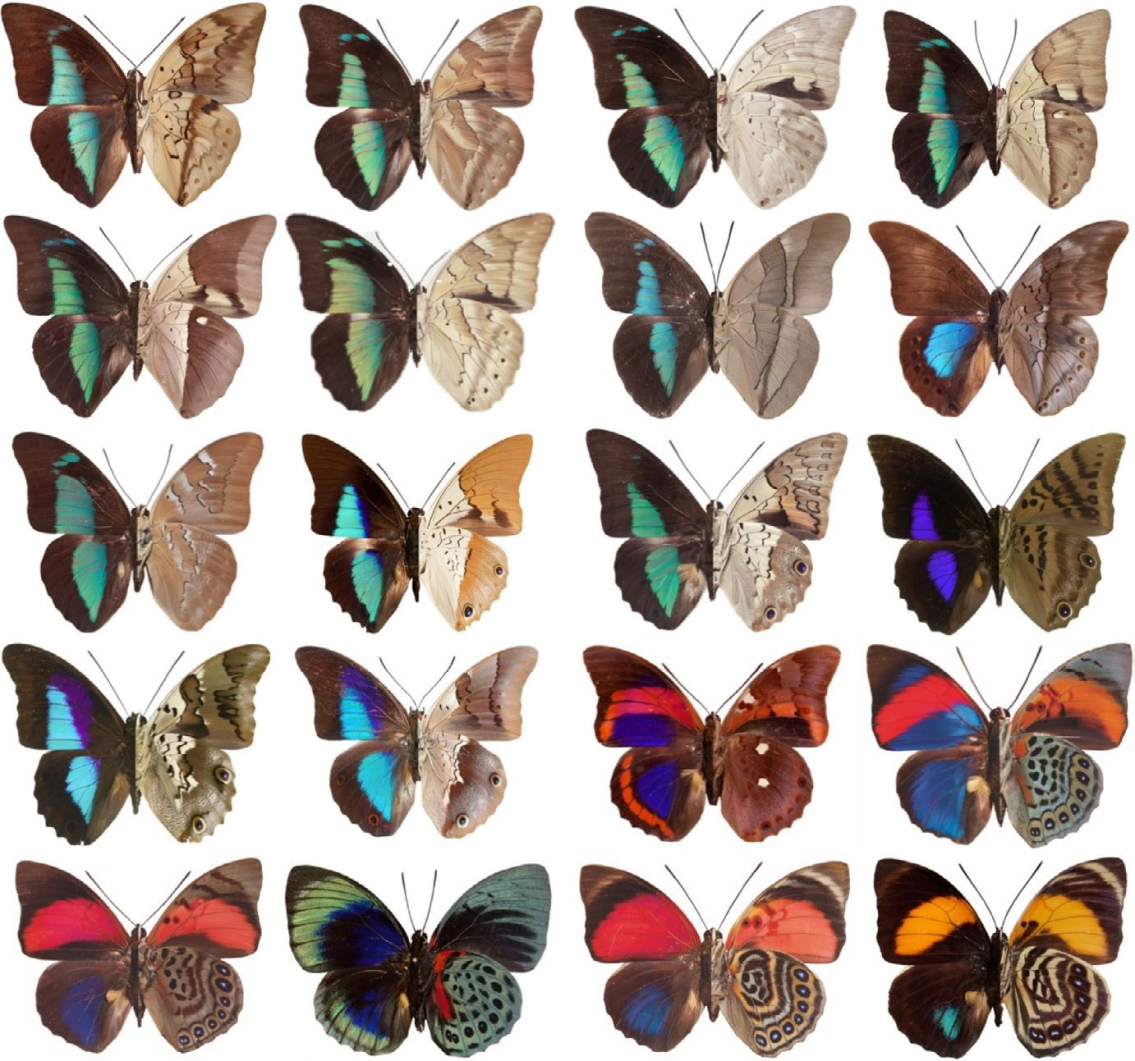
Example Preponini species. Butterflies shown (from left to right across each row): *Archaeoprepona licomedes, A. demophon, A. demophoon, A. amphimachus, A. meander, A. camilla, A. chalciope, A. priene, Mesoprepona pheridamas, Prepona dexamenus, P. laertes, P. werneri, P. gnorima, P. deiphile, P. praeneste, P. narcissus, P. aedon, P. hewitsonius, P. claudina, P. amydon*. Photos by P. S. Padrón and A. Warren

The Neotropical region contains approximately 45% of species in the family Nymphalidae, the most species‐rich family of butterflies (Chazot et al., 2020). The Nymphalidae contains such morphologically diverse groups of species that many currently recognized subfamilies were once treated as families. The family ranges from the often small and drab Satyrinae to some of the largest and most spectacularly colored butterflies, of which the Neotropical tribe Preponini (Charaxinae) is renowned as one of the most outstanding examples. Preponine butterflies inhabit the forest canopy and are characterized by robust bodies and erratic flight. They are distributed from Mexico to Argentina, with a species richness peak in the Amazon basin. Wing color patterns among Preponini species exhibit a dramatic transition from dorsally blue and ventrally brown to dorsally red/orange and ventrally multicolored (Figure 1). This color pattern variation explains why some *Prepona* species were long classified in a separate genus, *Agrias* (Ortiz‐Acevedo et al., 2017). The bright color patterns of preponines have attracted the attention of collectors and naturalists for over two centuries, resulting in hundreds of names for species within *Prepona* in particular (Lamas, 2004). Lamas (2004) recognized 22 species in the tribe, but subsequent additions and revisions have resulted 25 described species are now recognized, distributed in three genera *Archaeoprepona, Mesoprepona,* and *Prepona* (Ortiz‐Acevedo et al., 2017; Turrent Carriles et al., 2019). Nevertheless, no study to date has focused on understanding the role of color and biogeography on the diversification of this group.

In this study, we attempt to understand to what extent coloration and biogeographical events may have shaped the diversity of the tribe by quantifying color change and diversification rates, and estimating ancestral geographic ranges. We hypothesize that the main driver of Preponini evolution is color shifts, but biogeographical events in the last 30 Ma also likely influenced the origins of particular clades. Given that the members of *Prepona* show drastic changes in color patterns, we hypothesize that there might be a congruence between shifts in diversification and phenotypic evolutionary rates. Similarly, we expected that changes in diversification rates might be associated with major landscape changes, such as the Andes uplift and final closure of the Panama Isthmus.

## 2 MATERIALS AND METHODS

### 2.1 Phylogeny and divergence time estimation

We used DNA sequence data from Ortiz‐Acevedo et al. (2017). The final matrix comprises sequences from two mitochondrial and four nuclear gene fragments from 29 Preponini specimens including 24 of the 25 generally recognized species and five representatives of geographically and/or morphologically distinct populations of *Prepona deiphile* and *P. laertes* that we currently consider as distinct species (LlorenteBousquets, J. E. and E. Ortiz‐Acevedo unpublished data; Ortiz‐Acevedo, E. unpublished data; Neild, 1996; Turrent Carriles et al., 2019). We used as outgroup the sister tribe Anaeomorphini (Espeland et al., 2018). Out of Preponini and Anaeomorphini, our taxon sample excludes only the recently described *Prepona silvana* from western Mexico, apparently a close relative of what we refer to as *P. deiphile* (CA), and *P. "sahlkei*," treated by Neild (1996) as a distinct species from the Guianas but as synonym of *P. claudina claudina* by Lamas (2004), and whose status is currently uncertain (Table S1).

We partitioned the data by codon position a priori and then searched for the optimal partitioning scheme using the greedy algorithm in PartitionFinder v2.1.1 (Lanfear et al., 2012) (Table S2). Phylogenetic relationships and divergence times were inferred simultaneously using BEAST 2.4.5 (Bouckaert et al., 2014). The clock prior was set to an uncorrelated relaxed lognormal distribution, and site models were coestimated with the phylogeny using reversible jump by applying the bModelTest 0.3.3 plugin (Bouckaert and Drummond, 2017). Site models were unlinked, and tree models were linked. We ran three different analyses that differed in the number of molecular clocks used. The models consisted of (a) two molecular clocks, one for all mitochondrial partitions and the other for all nuclear partitions; (b) multiple clocks, one for all mitochondrial partitions and one for each nuclear partitions; and (c) multiple molecular clocks, one for each partition.

The tree prior was set to a Yule model. The birth rate prior was set to uniform with the lower boundary of 0 and upper boundary of 1,000, and the prior for the mean rate under the uncorrelated lognormal relaxed molecular clock (ucldMean) was set to a gamma distribution with alpha of 0.01 and beta of 1,000. We used a secondary calibration point for the common ancestor node for Preponini + Anaeomorphini assuming a normal prior distribution with mean at 32.9 Ma (95% credibility interval CI = 22.0–42.3 Ma) and sigma of 4.0 (Espeland et al., 2018). The value of sigma was selected to include the error associated with the primary dating study and incorporate the credibility interval estimated for the node Preponini + Anaeomorphini in the calibration (Forest, 2009). Three individual runs of 100 million generations were performed, sampling every 10,000 generations. The runs were combined in LogCombiner 2.4.5, and convergence was assessed in Tracer v1.6 (Rambaut et al., 2014). From the combined runs, we subsampled a total of 10,000 trees using LogCombiner to infer a maximum credibility tree in TreeAnnotator 2.4.5.

We used path sampling and stepping‐stone sampling to estimate the marginal likelihoods of the different molecular clock analyses (Baele et al., 2013) and used Bayes factors (Fan et al., 2011) to select the best analysis. We used 100 steps with a chain length of one million generations, and other settings were kept as default, and we tested convergence by examining that the average standard deviation of split frequencies fell below 0.01, visually inspecting the trace files in Tracer v1.6 (http://tree.bio.ed.ac.uk/software/tracer/), and checking that the effective sample size values for parameters were higher than 200.

### 2.2 Wing color pattern evolution

To reconstruct ancestral color patterns and estimate their rate of evolution, we photographed museum specimens deposited at the McGuire Center for Lepidoptera and Biodiversity, Florida Museum of Natural History (FLMNH‐MGCL) collection. We sampled on average 12 individuals per species, but some species, such as *Prepona amydon* (*n* = 61), had higher sampling due to their phenotypic diversity. We reduced possible color pattern variation caused by adverse collection storage conditions by selecting as recently collected specimens as possible (Figure S1).

Photographs in JPEG format were taken using a light box with a set of four daylight fluorescent Sylvania light bulbs and a Nikon D5300 camera body coupled with a Nikon 60 mm f/2.8G ED Auto Focus‐S Micro‐Nikkor Lens. We used a Kodak color separation guide and grayscale with a ruler included to calibrate the camera and the images before analysis. Color was measured at three independent locations on the forewing and over the forewing as a whole. Locations were delimited using wing veins, which delineate three homologous regions across species. The same region in the wing was measured consistently, irrespective of wing size or shape. The three forewing locations included the discal cell (Cell 1), and the regions delimited by the discal cell and the veins M_1_ and M_2_ (Cell 2), and CuA_1_ and CuA_2_ (Cell 3) (Figure S2). Those regions were selected to represent regions containing the most significant observed variation in color across the forewing and to control for differences in wing size.

On each of the wing regions and for the whole wing, we measured the mode of the red, green, and blue channels (RGB) and total RGB values using ImageJ (Abramoff, 2004; Rasband, 1997; Schneider et al., 2012). In total, we measured color as a set of 16 traits. We preferred the mode over the mean because in a skewed distribution, it better describes where the bulk of the density is concentrated, while the mean may deviate from the mode because of large numbers in the tails of the distribution. Since we wanted to test for differences in evolutionary rates and the signature of evolution in the three different cells and the overall wing, we did not attempt to reduce the variable set by means of principal component analysis. Additionally, since Preponini consists of three genera in two clades, one mainly blue and the other blue and red, we wanted to evaluate the rates of evolution of the different color channels independently.

To test for changes in evolutionary rates in color across lineages, we used the function rjmcmc.bm in the package "geiger 2.0" (Harmon et al., 2008) in R, which implements a Bayesian approach using a reversible jump Markov chain Monte Carlo (rjMCMC) process to compare among four different models of changes in evolutionary rates (Eastman et al., 2011). The first was a single rate Brownian motion (BM) model in which phenotypic rate evolution was constant across the tree. Next, we fitted a relaxed BM model (rBM1) in which evolutionary rates were allowed to shift multiple times across trees. Third, we kept evolutionary rate constant (no shift), but traits were allowed to pulse rapidly, representing jumps in the mean of the trait (jump‐BM). Last, we combined rBM1 and jump‐BM to allow phenotypic rates to shift several times and the mean trait to jump across the tree (jump‐rBM, Eastman et al., 2011). Before running the models, we calibrated the rjMCMC and estimated that the most reasonable proposal width to initiate sampling of the Markov chain was eight for every model evaluated (see Eastman et al., 2011).

Model selection was performed using the Akaike information criterion (AIC) for MCMC samples (Raftery et al., 2007). The difference in AIC (ΔAIC) between models was used to select the best model. We assumed a model to be significantly better than another if ΔAIC > 2. In the case in which ΔAIC < 2, we selected the simplest model in the following order: (a) BM, (b) rBM1, (c) jump‐BM, and (d) jump‐rBM. We did not perform model averaging in cases in which −2 < ΔBIC < 2, because it has been suggested to be misleading (Taper & Ponciano, 2016).

For each model in each cell and measurement, we ran two chains each for 1,250,000 generations sampling every 1,000. We assessed convergence of the MCMC by inspecting the trace of each parameter and estimating the effective sample size. Finally, we used maximum‐likelihood ancestral state reconstruction to visualize trait evolution (Felsenstein, 2004) using the ace function in "ape" (Paradis et al., 2004) in R. The shifts in R and B rate on the same branch might be due, however, to more or less rapid fluctuations between red and blue, or alternatively, they might be due to more or less rapid fluctuations in lightness. Since we hypothesize that changes were mainly due to changes in hue from red to blue rather than lightness, to account for the above‐mentioned issue, we computed a lightness‐independent index by regressing the RGB values against lightness and repeated the analyses using the residuals of this regression (Figure S3).

### 2.3 Diversification dynamic estimation

To investigate whether diversification rates varied through time, we used a series of likelihood‐based diversification models in the R package RPANDA (Morlon et al., 2016). One of the models implemented in RPANDA estimates the likelihood that speciation and extinction are constant or variable through time. Accordingly, we tested a combination of models in which we allowed speciation and extinction to be either constant or variable following a linear or an exponential model (see Table 1 for model specification and results for details on linear models). We compared the models using AIC.

**Table 1:** Model specifications for the RPANDA diversification rate analyses

	Lambda	Mu	Lambda		Mu		Loglik	AIC	ΔAIC
			Intercept	Coefficient	Intercept	Coefficient			
Time models	Constant	Zero	0.12		0		−90.9	183.8	0.4
	Exponential	Zero	0.15	−0.03	0		−89.9	183.8	0.4
	Constant	Constant	0.13		−0.04		−90.7	185.4	2
	Exponential	Constant	0.15	−0.03	>0.0001		−89.9	185.8	2.4
	Exponential	Exponential	0.15	−0.03	>0.0001	0.03	−89.9	187.8	4.4
	Constant	Exponential	0.14		0.01	0.11	−89.6	185.2	1.8
Andes models	Exponential	Zero	0.02	0.49	0		−89.7	183.4	0
	Exponential	Constant	1.14	−0.43	0.18		−94.4	194.8	11.4
	Constant	Exponential	0.14		0.72	−0.93	−89.7	185.4	2
Panama Isthmus models	Exponential	Zero	0.06	0.8	0		−90.2	184.4	1
	Exponential	Constant	0.04	1.23	>0.0001		−90.7	187.4	4
	Constant	Exponential	0.11		>0.0001	0.7	−90.9	187.8	4.4

In addition to the RPANDA time‐dependent diversification analyses, we used alternative methods to support the estimation of diversification rates for the tribe. Overall, we estimated diversification rates using the constant‐rates test (CRT; Pybus & Harvey, 2000), Magallón and Sanderson's estimator (Magallon & Sanderson, 2001), and Bayesian analysis of macroevolutionary mixtures (BAMM; Rabosky, 2014) and MEDUSA (Alfaro et al., 2009), and visualized the results with a lineage‐through‐time plot (LTT). Furthermore, we used a recently developed method, which estimates unbiased speciation and extinction rates based on LTT (Louca & Doebeli, 2017; Louca & Pennell, 2020). Details about these diversification analyses are provided in the Appendix S1.

### 2.4 Trait‐dependent diversification analyses

To test whether Preponini lineage diversification has been dependent on phenotypic evolution (i.e., color pattern), we used the equal‐splits with simulated null model (ES‐sim) method (Harvey & Rabosky, 2018). ES‐sim employs a tip and rate correlation technique to test for the significance of the correlation between trait values at the tips and the phylogeny's branching pattern in the absence of an evolutionary model. Harvey and Rabosky (2018) showed that for phylogenies with ~50 species, ES‐sim performed better in the type I error rate and had comparable power to QuaSSE (Quantitative State Speciation and Extinction, FitzJohn, 2010), a method commonly used to jointly model diversification and the change in continuous traits. In addition, ES‐sim is useful for small phylogenies with few and slight changes in diversification rate, which might be the case in our study (Harvey & Rabosky, 2018). As a result of the ES‐sim test, we obtained (a) a Pearson's correlation coefficient (*ρ*), which quantifies the strength of the relationship between traits and each lineage's diversification rate; and (b) the simulation‐based two tailed probability value (*p*‐value), which allows comparison of the data to a null hypothesis.

### 2.5 Biogeographical‐dependent diversification analyses

To test whether the Andean uplift or closure of the Panama Isthmus influenced Preponini diversification rates, we used a similar likelihood approach as for time‐dependent diversification analyses implemented in RPANDA. In this case, we evaluated the fit of six models allowing speciation and extinction rates to be dependent on the paleoelevation of the Andes (three models) or the degree of connectivity between Central and South America (three models; Morlon, 2014; Morlon et al., 2011; see Table 1).

We used Andean paleoelevation from present time until 32 Ma based on the database provided by Lagomarsino et al. (2016) and information from Garzione et al. (2008). The closure of the Panama Isthmus was estimated using reports of migration rates for several groups of organisms by Bacon et al. (2015). In the latter study, they reported that migration rates between the two continents increased sequentially at four points in time, 41.1, 23.7, 8.7, and 5.2 Ma. We used these migration rate estimates to compute the probability of observing at least one migration event in each time period (i.e., 50–41.1, 41.1–23.7, 23.7–8.7, 8.7–5.2, and 5.2–present). We then used the cumulative probability through time to observe at least one migration event per million years as a proxy of the degree of connectivity between land masses (Table S3).

### 2.6 Estimation of ancestral ranges

We used a Microsoft Access database to compile locality data from 4,050 Preponini specimens deposited at five butterfly collections: the McGuire Center for Lepidoptera and Biodiversity, Florida Museum of Natural History, University of Florida, National Museum of Natural History, Smithsonian Institution, American Museum of Natural History, Instituto Alexander von Humboldt, and the Instituto de Ciencias Naturales— Universidad Nacional de Colombia Sede Bogotá. We georeferenced localities using Google Earth, literature, and published/unpublished gazetteers, and cleaned the database to remove erroneous and imprecise localities. Our final database contained 1,121 locality records for the 31 taxa in consideration.

We used the R package BioGeoBEARS 1.1.1 (Matzke, 2018) to estimate the ancestral range of Preponini under the dispersal–extinction–cladogenesis (DEC) model (Ree & Smith, 2008) and a maximum‐likelihood implementation of the dispersal–vicariance analysis (DIVALIKE) model (Ronquist, 1997). We did not include models with the parameter J since they seem less relevant to continental settings and have been shown to be difficult to compare statistically to other non‐nested models (Ree & Sanmartín, 2018). We tested eight different hypotheses to evaluate the influence of different biogeographical events and distance among areas in the evolutionary history of Preponini (see Table 2 for details).

**Table 2:** Biogeographical hypotheses tested in the study

Code	Hypotheses	Restriction to dispersal		
		Distance	Panama	Andes
Ho1	Null	na	na	na
	There is no restriction to dispersal.			
Ho2	Restriction to dispersal by distance	yes	na	na
	Geographic events do not have any influence on the ancestral range reconstruction. Dispersal is only restricted by geographic distance.			
Ho3	Restriction to dispersal by Panama	na	yes	na
	The gradual closing of the Panama Isthmus is the only geographic event that influenced Preponini dispersal.			
Ho4	Restriction to dispersal by Andes	na	na	yes
	The gradual uplift of the Andes mountain range is the only geographic event that influenced Preponini dispersal. The Andes uplift generated a reconfiguration of the Amazonian lowland landscape by aiding the formation of the Pebas system and subsequently the Acre system.			
Ho5	Restriction to dispersal by distance + Andes	yes	na	yes
	Preponini dispersal was influenced by a combination of geographic distance and the uplift of the Andes mountain range.			
Ho6	Restriction to dispersal by Distance + Panama	yes	yes	na
	Preponini dispersal was influenced by a combination of geographic distance and the closing of the Panama Isthmus.			
Ho7	Restriction to dispersal by Panama + Andes	na	yes	yes
	Preponini dispersal was influenced by a combination of both the closing of the Panama Isthmus and the uplift of the Andes mountain range.			
Ho8	Restriction to dispersal by distance + Andes + Panama	yes	yes	yes
	Preponini dispersal was influenced by a combination of geographic distance, the closing of the Panama Isthmus, and the uplift of the Andes mountain range.			

Abbreviations: na, not applicable.

Briefly, the hypotheses account for the influence of the closing of the Panama Isthmus, the formation of the Andes, and distance among eight geographic areas in the Neotropics. We coded the areas as Central America, Caribbean, Chocó and Caribbean lowlands, Western Andes, Eastern Andes, Amazon (including Chaco and Cerrado), Guianas, and Atlantic Forest, based on NatureServe's classification of the Neotropics into "Ecological Systems" (Josse et al. 2003). We chose not to split the Amazon into smaller regions, as has been done in some other studies, because most species present in the Amazon are widespread within this area. Since one of our objectives was to test for the influence of major biogeographical events, we designated four time slices representing different paleogeological stages. The four time slices used in our analyses were as follows: (a) 32–23, (b) 23–10 Ma, (c) 10–7 Ma, and (d) 7 Ma–present, which represent the gradual formation of the connection between South and Central America and Andean uplift (Bacon et al., 2015; Condamine et al., 2012; Montes et al., 2015). We allowed the probability of movement across areas to change in time, accounting for geographic position, for distance and for barriers to dispersal, and penalized accordingly (Tables S4–S11). We evaluated the effect of distance by penalizing dispersal probabilities with a factor proportional to the distance among the centroids of the areas (Tables S5, S8–S9, S11).

Both DEC and DIVA are biased toward estimating widespread ranges in deep nodes (Buerki et al., 2011; Clark et al., 2008; Matzke, 2014; Ree & Smith, 2008). In an attempt to avoid this potential bias, the maximum number of areas any ancestor may occupy was set to six, since this is the maximum number of areas observed to be currently occupied by any single Preponini species (Ronquist & Sanmartin, 2011; e.g., *Archaeoprepona demophoon*). We reduced the number of potential ranges in the reconstruction by including only geographic ranges with adjacent areas. This resulted in 113 potential ranges from the 248 possible combinations. To identify the hypotheses with strongest support, we compared AIC among the eight hypotheses and two possible models.

## 3 RESULTS

### 3.1 Phylogeny and divergence time estimation

**Figure 2:**
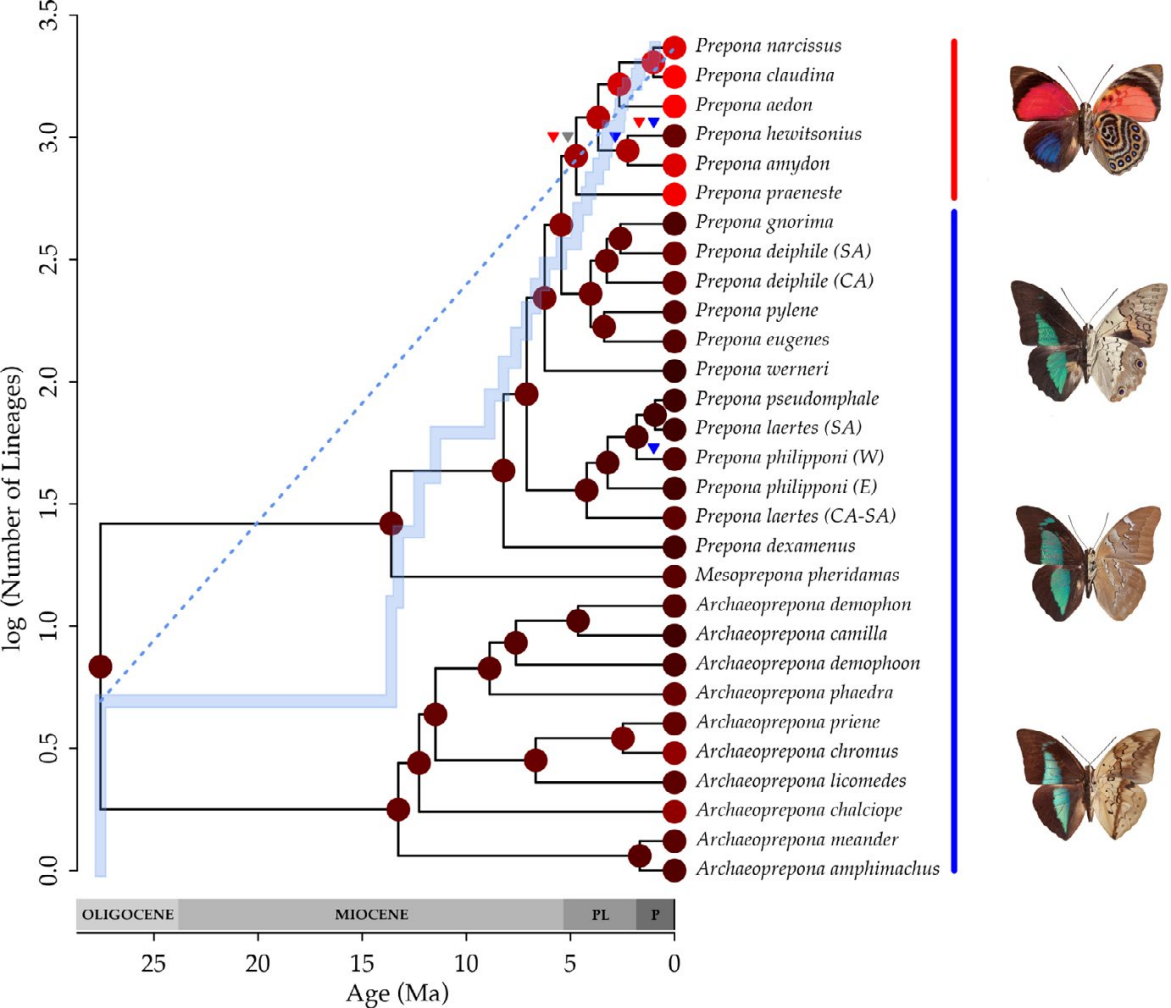
Ancestral reconstruction of the mode RGB red channel for the Cell 1 and lineage‐through‐time plot (solid blue line). Blue dotted line denotes expectation of lineage accumulation under a pure birth process. Red, blue, and gray triangles highlight branches where a jump in color was identified for the blue, red, and total channels in Cells 1 and 2 (see text for details). Vertical color bars denote the red and blue Preponini clades. Abbreviations follow: PL: Pliocene, P: Pleistocene. Butterflies shown (from top to bottom): *Prepona claudina, P. laertes, Mesoprepona pheridamas,* and *Archaeoprepona licomedes*. Photos by P. S. Padrón

**Figure 3:**
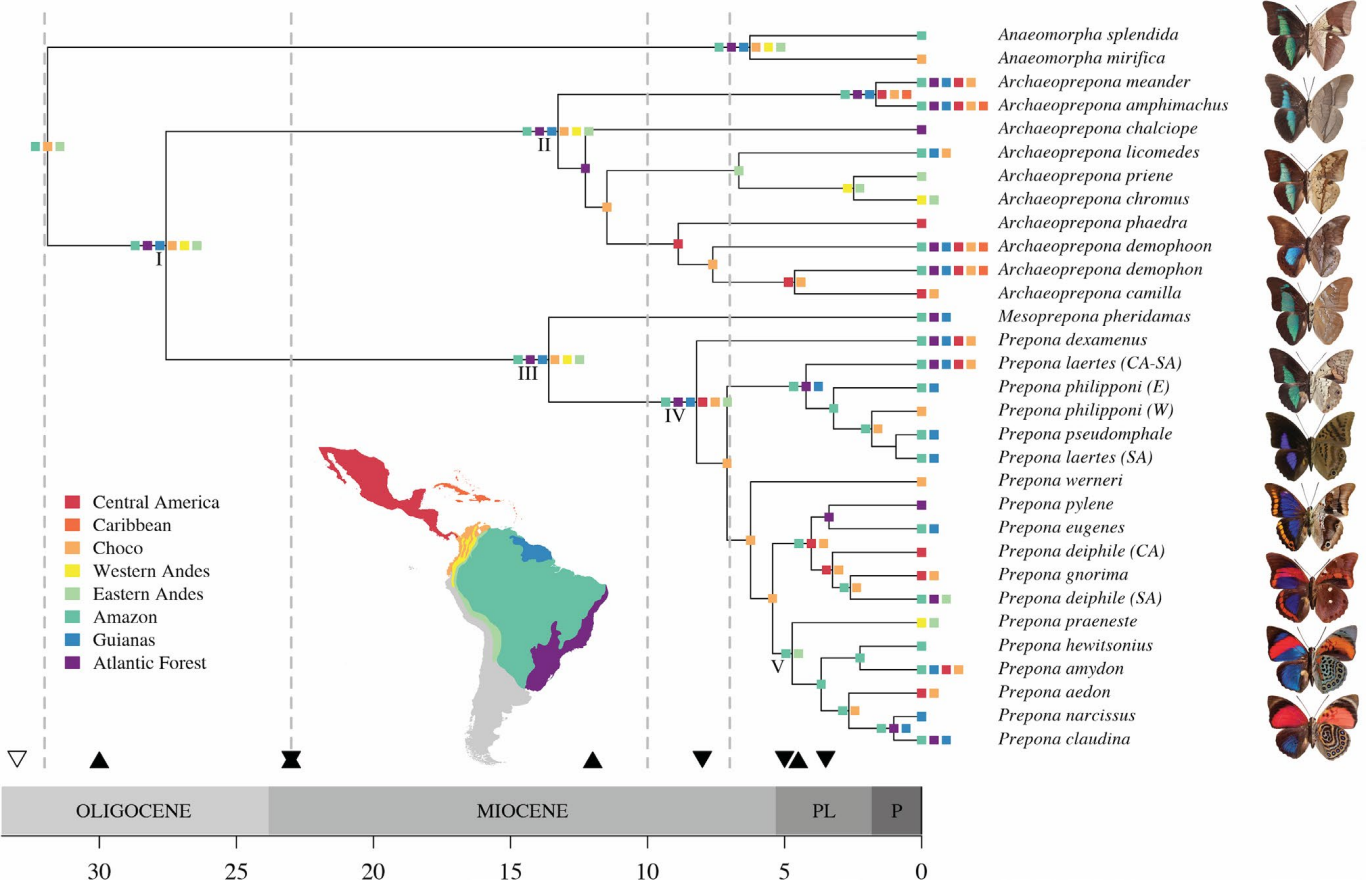
Biogeographical range estimates for the tribe Preponini based on the DEC model. Dotted gray lines delimit the time slices used in the stratified analysis. Upward black triangles denote periods of increased Andean uplift (Hoorn et al., 2010), downward black triangles denote periods of increased biological migration between Central and South America, and downward white triangle denotes initial faunal exchange between Central and South America at ~41 Ma (Bacon et al., 2015). Roman numerals in nodes refer to the main text. Abbreviations follow: PL: Pliocene, P: Pleistocene. Butterflies shown (from top to bottom): *Archaeoprepona meander, A. chalciope, A. licomedes, A. priene, Mesoprepona pheridamas, Prepona laertes, P. werneri, P. deiphile, P. praeneste, P. narcissus, P. claudina*. Photos by P. S. Padrón and EOA

The three analyses with different molecular clocks were equally supported (BF = ~1; Tables S12 and S13). The phylogenetic reconstruction is mostly congruent with published topologies and has good support overall (Figures 2 and 3, Figure S4) (Ortiz‐Acevedo et al., 2017; Ortiz‐Acevedo & Willmott, 2013). The origin of Preponini was estimated at ~27.6 Ma in the Oligocene, that of *Archaeoprepona* and *Mesoprepona* was estimated at ~13 Ma in the mid‐Miocene, and that of *Prepona* was estimated at ~8 Ma in the late Miocene (Figures 2 and 3, Figure S4). The red *Prepona* clade originated in the early Pliocene, ~5 Ma.

### 3.2 Wing color pattern evolution

Although the overall signature of evolution suggests constant phenotypic evolutionary rates, we identified two jumps in evolutionary rate in the red channel Cell 1 in the red *Prepona* clade: the first one at the base of the red *Prepona* clade and the second along the branch leading to *Prepona hewitsonius* (Table 3, Figure 2, Figure S5). Similarly, our analyses identified one jump in the total RGB of Cell 1 at the base of the red *Prepona* clade. The blue channel in Cell 2 showed 3 jumps (posterior probability > 0.5), located at the base of the *P. hewitsonius* + *P. amydon* clade, at the branch leading to *P. hewitsonius*, and at the tip leading to *P. philipponi (W)* (Figure 2, Figure S5). The analysis using the lightness‐independent index yielded similar results (Table S14, Figure S6), so we only show and discuss the results using the raw RGB data.

**Table 3:** Evolutionary rates for the mode color of the different areas of the wing measured

Measurement	*α*	Jumps	***σ*** ^2^	LnL	AIC	Model
Wing						
Total	82.96		53.02	−129.09	259.98	BM
Red	128.85		186.52	−157.23	320.82	BM
Green	73.66		20.68	−118.07	238.19	BM
Blue	83.12		232.9	−168.62	345.61	BM
Cell 1						
Total	64.5	1.37	35.71	−126.89	265.92	jump‐BM
Red	92.43	2.19	49.92	−140.84	292.55	jump‐BM
Green	55.6		45.93	−128.91	259.38	BM
Blue	40.23		48.33	−138.92	279.31	BM
Cell 2						
Total	86.93		41.99	−130.93	263.83	BM
Red	137.32		137.37	−154.44	313.48	BM
Green	76.6		41.53	−129.56	260.75	BM
Blue	47.66	3.85	17.55	−131.46	295.53	jump‐BM
Cell 3						
Total	99.23		69.91	−136.34	274.35	BM
Red	128.92		181.99	−156.98	319.92	BM
Green	85.41		54.28	−134.38	270.41	BM
Blue	107.52		182.32	−161.35	328.75	BM

Note

*α* is defined as the estimated value of the character at the root and ***σ*** ^2^ as the evolutionary rate (or variance).

### 3.3 Diversification dynamic estimation

The RPANDA analyses identified the time‐dependent model with constant speciation and no extinction, and with exponentially varying speciation, as the best models (Table 1). The first model suggests speciation rate to be 0.12 LogSpecies per Ma. In the second model, which has support similar to the constant diversification model, speciation increases through time from 0.05 at 32 Ma to 0.15 LogSpecies per Ma in the present. Models assuming linearly varying speciation and extinction did not converge; consequently, we do not show the results here. Most other complementary diversification analyses agree with the constant diversification rate model, except MEDUSA (Appendix S1). Moreover, estimates of unbiased speciation and extinction rates were similar to those obtained using the maximum‐likelihood approach in RPANDA (Appendix S1). Furthermore, despite recent critiques of approaches for estimating speciation rates, all models that we tested resulted in similar parameter estimates of between 0.12 and 0.17 LogSpecies per Ma (i.e., 1.12 and 1.18 species per Ma).

### 3.4 Trait‐dependent diversification analyses

We found that diversification rate was not dependent on any of the color traits. We found that the absolute value of Pearson's rho was smaller than 0.54 for all cells and the full wing, suggesting low correlation between the equal splits (ES) and trait values (Table 4). Furthermore, none of the Pearson's rho was significantly different from the null expectation.

**Table 4:** Trait‐dependent diversification analysis for the color traits

Measurement	*ρ*	*p*‐value
Wing		
Total	0.15	0.81
Red	0.25	0.67
Green	−0.41	0.43
Blue	0.25	0.68
Cell 1		
Total	0.43	0.39
Red	0.33	0.56
Green	0.00	1.00
Blue	0.54	0.18
Cell 2		
Total	0.04	0.95
Red	−0.11	0.87
Green	−0.24	0.70
Blue	0.33	0.56
Cell 3		
Total	0.17	0.78
Red	0.34	0.55
Green	−0.39	0.45
Blue	0.09	0.89

### 3.5 Biogeographical‐dependent diversification analyses

Diversification models accounting for the influence of biogeographical events on the diversification rates of Preponini suggest that speciation and both the Andean uplift and the closure of the Panama Isthmus were exponentially and positively correlated through time (Table 1). In fact, the model with exponentially varying speciation rate depending on Andean uplift had a slightly lower AIC than the time‐dependent models (Table 1).

### 3.6 Estimation of ancestral ranges

BioGeoBEARS analyses identified DEC as the best model with restriction to dispersal and an influence of the closure of the Panama Isthmus and Andean uplift (Ho7) as the hypothesis with strongest support (Figure 3, Table 5). The tribe most likely originated in South America (node I in Figure 3, Figure S7). The genus *Archaeoprepona* retained its ancestor's range of origin (node II in Figure 3); other ranges with high probability also suggest a continental America origin (Figure S7). Similarly, *Mesoprepona + Prepona* likely originated in South America (node III; Figure 3); the other probable regions of origin comprise broad ranges as for its sister genus (Figure S7). The origin of *Prepona* was most likely in mainland South America, excluding high‐elevation habitats of the western Andes (node IV; Figure 3). Finally, the clade that corresponds to the transition of color patterns from mostly blue to red *Prepona* species was found to have most likely originated in the Amazon and Eastern Andes (node V; Figure 3, Figure S7); the other ranges with high probability are narrow, where Andes, Amazon, and Chocó are also likely regions of origin (Figure S7).

**Table 5:** BioGeoBEARS results for the models and hypotheses tested in the study

Model	Hypothesis	LnL	# Parameters	*d*	*e*	*x*	AIC	ΔAIC
DEC	Ho1	−147.54	2	0.06	0.05	na	299.09	15.13
DEC	Ho2	−284.93	3	0.20	0.42	1	575.87	291.91
DEC	Ho3	−148.30	2	0.07	0.03	na	300.59	16.63
DEC	Ho4	−144.13	2	0.16	0.10	na	292.27	8.31
DEC	Ho5	−217.62	3	0.20	0.42	1	441.23	157.27
DEC	Ho6	−263.13	3	0.20	0.42	1	532.26	248.30
**DEC**	**Ho7**	**−139.98**	**2**	**0.22**	**0.10**	**na**	**283.96**	**0.00**
DEC	Ho8	−164.86	3	0.20	0.42	1	335.73	51.77
DIVA	Ho1	−148.10	2	0.07	0.05	na	300.21	16.25
DIVA	Ho2	−285.73	3	0.20	0.42	1	577.46	293.50
DIVA	Ho3	−148.96	2	0.08	0.03	na	301.93	17.97
DIVA	Ho4	−145.09	2	0.14	0.06	na	294.17	10.21
DIVA	Ho5	−216.76	3	0.20	0.42	1	439.53	155.57
DIVA	Ho6	−263.44	3	0.20	0.42	1	532.89	248.93
DIVA	Ho7	−143.29	2	0.18	0.05	na	290.57	6.61
DIVA	Ho8	−161.44	3	0.20	0.42	1	328.88	44.92

Note

Bold text references the best model identified for the data. *d*: dispersal, *e*: extinction, *x*: distance, na: not applicable.

## 4 DISCUSSION

In our study, we hypothesized that shifts in color patterns and/or biogeographical events have contributed to Preponini diversification. Despite finding high rates of wing color evolution, mostly in the red *Prepona* clade (Figure 2; node V in Figure 3), we found that diversification rates in Preponini were not dependent on color evolution. However, major landscape reconfiguration, happening through most of the tribe's evolutionary history, did seem to influence speciation rates and distribution patterns (Table 1 for models of biogeography‐dependent diversification analysis results). The Panama Isthmus and the uplift of the Andes mountain range together contributed most to the distribution patterns of extant Preponini. Cladogenesis happened mostly through dispersal to new ranges or splitting of broadly distributed taxa, as suggested by the Dispersal Extinction Cladogenesis model (DEC; Table 5), and Preponini likely originated within South America.

### 4.1 Wing color pattern evolution

During the course of their evolution, *Prepona* underwent drastic changes in the color pattern of both wing surfaces (Figures 2 and 3). These color patterns were the key features used by previous taxonomists to classify *Prepona* species in two genera. The former genus *Agrias* contained yellow, orange, blue, and red species, and *Prepona*, as delimited previously, contained mostly blue species. The former group being nested within the latter is supported by molecular and morphological data (Ortiz‐Acevedo et al., 2017), while color patterns apparently transitioned rapidly from blue to red (Figure 2), resulting in *Prepona* containing butterflies with such strikingly different coloration patterns (Figures 1, 2, 3).

Our results suggest that there is stronger change in phenotype for red and blue RGB channels (Table 3) than for green and total RGB. The changes identified are mostly jumps located around the red *Prepona* clade (Figure 2). The analysis consistently identified the jump from blue to red in Cell 1 with no shift in the rate of evolution. Despite the fact that the red *Prepona* clade is so notably different in color pattern compared with the rest of the tribe's members, the evolutionary rate of phenotypic change after the jump remained similar to that in blue clades. These jumps are consistent with previous findings that drastic changes in color patterns of butterfly wings and other organisms have a relatively simple genetic basis and can appear relatively fast in evolutionary time (Nadeau et al., 2016; Reed et al., 2011). Differential genetic expression in particular regions of the wing is also responsible for localized changes (Brakefield et al., 1996; Nadeau et al., 2016; Oliver et al., 2012), which might be a plausible explanation for the results we found in Cell 1. These localized changes are not always visible under analysis of the entire wing; thus, isolating those regions that are likely to change quickly allowed us to detect the jumps in phenotype.

Jumps and shifts in the blue channel are restricted to the tips of the phylogeny and might be associated with recent speciation events. For example, we identified two jumps in the blue channel of Cell 2 (Figure 2): (a) in the branch leading to *Prepona amydon + P. hewitsonius* and (b) in *P. hewitsonius*. These species are phenotypically extremely diverse in forewing coloration, ranging from dark, deep blue to light, bright blue, even within subspecies (Figure S8). We also identified a change in the Cell 2 blue channel at the branch leading to *Prepona philipponi (W)*. *Prepona philipponi (W)* has a different light blue tone compared with *P. philipponi (E)* (Figure S9), but with only a single studied individual of the former taxon, this result needs corroborating with further samples.

A potential explanation for the dramatic change (jump) in color patterns within Preponini is involvement in mimicry rings with genera such as *Callicore* and *Asterope* (Nymphalidae, Biblidinae), which have remarkably similar color patterns on both wing surfaces (Descimon, 1977; Jenkins, 1987; Figure S10). Preliminary geographic distribution data show a correspondence in coloration and distribution among *Prepona*, *Callicore,* and *Asterope* (Ortiz‐Acevedo, E. unpublished data). The inferred origin of the red clade in *Prepona* partially supports this hypothesis, since both *Callicore* and *Asterope* are predominantly Andean and Amazonian groups. In particular, the red *Prepona* showed no change in the Amazonian ancestral range as it underwent speciation (Figure 3). Classical Müllerian and Batesian mimicry is plausible since species in both groups feed on toxic plants, including the families Sapindaceae and Erythroxylaceae, but whether they sequester plant toxins as caterpillars and retain them after emerging as adults is still unknown, as is their palatability. Alternatively, escape mimicry is also a possible explanation, since all species involved are fast flyers (Pinheiro & Freitas, 2014). Fast and erratic flying has been suggested as an antipredator defense mechanism, where predators learn to avoid these species due to the high‐cost–low‐benefit trade‐off (Ruxton et al., 2004; van Someren & Jackson, 1959).

### 4.2 Diversification of preponines

In studies of other Neotropical butterfly groups, natural history traits and paleoclimatic events have been shown to shape diversification rates by influencing speciation and/or diversification (Chazot et al., 2020; De‐Silva et al., 2016; Sahoo et al., 2017). This seems to be the case for Preponini as well. We found support for exponentially varying speciation rates, similar to results in Neotropical Nymphalidae (Chazot et al., 2020). Extinction in the three top candidate models was a priori fixed to zero, as estimated for larger Neotropical butterfly clades during the same geological time (Chazot et al., 2020). The current diversification rate estimated by our likelihood analysis (0.15) is within the range of diversification rates estimated for other butterfly groups (Chazot et al., 2020; Peña & Espeland, 2015). We acknowledge, however, that the models with constant speciation have slightly weaker support than exponentially increasing speciation rate models. We thus cautiously interpret our results, favoring the model with the lowest AIC.

Our analyses are most consistent with an exponential increase in speciation promoted by Andean uplift (Table 1). In fact, this biogeographically dependent model has slightly higher support than the time‐dependent models. Species distributions in *Prepona* support the fact that the uplift of the Andes might have promoted higher speciation rates. There are several examples within *Prepona* of allopatric sister clades on either side of the Andes, which are consistent with the uplift of the Andes as a major vicariant event influencing species formation (Figure 3). A similar result has been found in many other organisms, both lowland and highland, and is hypothesized to result from the ecological opportunity created by emergence of new niches and the restriction of dispersal across the Andes (Smith et al., 2014). Our ancestral range estimates show that, after the two last pulses in Andean uplift, lineages mostly contracted in their ranges leading to allopatric sister clades. For example, the ancestor of the clade containing *Archaeoprepona licomedes*, *A. priene,* and *A. chromus* most likely had an eastern Andean distribution, while its sister clade had a probable Central American ancestor (Figure 3). Also, the clade containing all *Prepona* species except *P. dexamenus* originated from a Chocoan ancestor, and one daughter lineage retained its ancestral range, while the other dispersed to the Amazon, Guianas, and Atlantic forest (Figure 3). The origin of many recent Preponini species dates to a time of intensified Andean uplift, in which the Pebas system started to drain and the Amazon river reached its current configuration, aided by the formation of the Acre system (Hoorn et al., 2010).

A recent study showed that phylogenetic trees alone do not have enough information to tease apart different evolutionary scenarios since a particular topology can either result from high speciation and constant extinction or decreased extinction and constant speciation (Louca & Pennell, 2020). Consequently, we would expect similar support for these alternative scenarios. Here we show, however, that support for models with constant speciation and no extinction or exponentially varying speciation with no extinction is much stronger than for models with constant speciation and varying extinction under an evidential statistics framework (Taper & Ponciano, 2016). In a recent comment to Louca and Pennell (2020), Morlon et al. (2020) advocated for the kind of hypothesis‐driven approach that we adopt here, suggesting that the conclusion that speciation and extinction rates are not identifiable parameters does not apply to all cases. Morlon et al. (2020) point out that the fact that there are many congruent diversification models for the same tree topology does not pose a direct challenge to the hypothesis‐driven approach used here.

### 4.3 Origin and biogeographical patterns

Although the high Amazonian species richness of the tribe would suggest an out‐of‐the‐Amazon biogeographical model, the tribe Preponini originated from a widespread South American ancestor at ~27.5 Ma in the early Oligocene. By this time, South America, dominated by forests (Strömberg et al., 2013), was detached from Antarctica and moving north toward North America (Axelrod et al., 1991). This dating is consistent with other studies that used independent datasets (Peña & Wahlberg, 2008; Wahlberg et al., 2009). Members of Preponini likely dispersed, colonized, and diversified in new niches in Central America as the bridge between land masses became more continuous at ~23 Ma (Bacon et al., 2015; Montes et al., 2015). This event has been demonstrated to have played a major role in the diversification of multiple groups of organisms (Bacon et al., 2015).

It has been suggested that some of methods for ancestral range reconstruction are biased toward estimating widespread ancestral ranges (Buerki et al., 2011; Clark et al., 2008; Matzke, 2014; Ree & Smith, 2008), as we found in the ancestor of Preponini. To reduce this bias, we restricted the ancestral estimate to contain only six of the eight areas considered, as observed in the most widespread extant Preponini species (Ronquist & Sanmartin, 2011, i.e., *Archaeoprepona amphimachus, A. demophoon, and A. demophon*). In addition, climatic changes accompanying the evolution of Preponini make biological sense in light of our inferred ancestral range estimate, as we discuss below.

Early speciation events in the tribe are characterized by range maintenance (~13 Ma; nodes II and III; Figure 3). Subsequently, the ancestor of *Archaeoprepona* contracted in range, becoming restricted to the Atlantic forest region and subsequently dispersing to the Chocó. The transition from Atlantic Forest to Chocó happened through an anagenetic expansion, including all South American areas except Western Andes, and a subsequent range contraction (Table S15). The restriction of the ancestor of *Archaeoprepona* to Atlantic forest is coincident with a period in which tropical forests in South America were likely reduced by a decrease in global temperature (Kürschner et al., 2008; Pound et al., 2011). The late Miocene was probably characterized by grassland habitats, as suggested by high diversification of hoofed animals and changes in their dentition (Kürschner et al., 2008). Preponini species are not currently known to inhabit these grassland habitats. The subsequent dispersal to the Chocó follows an increase in global temperature and the reappearance of widespread tropical forests (Kürschner et al., 2008; Pound et al., 2011).

After the colonization of Chocó tropical forests, *Archaeopreopona* dispersed to central America at ~8 Ma during periods of intensified biological migration between Central and South America (Bacon et al., 2015). From this point forward, high geologic activity in South America during the Pliocene (Hoorn et al., 2010) likely promoted dynamism in current ranges in which some species became widespread (clades containing *Archaeoprepona demophoon* and *A. amphimachus*), while others became restricted (e.g., *A. chromus* and *A. priene*) to small geographic areas.

Conversely, *Prepona* + *Mesoprepona*, which originated from a South American ancestor, show contrasting patterns of range evolution. *Mesoprepona*, a monotypic genus, shows a reduction in its distribution range and became restricted to eastern South America. In contrast, the relatively species‐rich *Prepona* shows early diverging clades to have dispersed to a larger area, including South and Central America, around a time of increased migration between these two continents (Bacon et al., 2015). The early branching *Prepona laertes* clade was found to be initially restricted to eastern South America, with subsequent dynamism in range contraction/expansion as it underwent speciation events. More recent clades were found to have contracted their ranges at ~7 Ma, becoming restricted to the Chocó region, while the Amazon region suffered landscape reconfiguration resulting from the transition of the Pebas system to the Acre system.

The subsequent speciation events show the following: (a) range maintenance in the restricted *Prepona werneri*, (b) range expansion followed by contraction in the *P. pylene* and *P. deiphile* clade, and (c) slight range expansion followed by contraction in the red *Prepona* clade (node III, Figure 3). The dynamic range evolution in these clades happened in the early Pliocene ~5 Ma, which is characterized by landscape reconfiguration due to a strong activity in Andean uplift (Hoorn et al., 2010; Figure 3). This intensified final uplift of the Andes likely allowed the earliest divergent red *Prepona* to shift from lowland–highland distribution and become restricted to higher elevation habitats, while the ancestor of the remaining red *Prepona* species became restricted to the Amazonian lowlands. Current distribution ranges of the taxa in this clade show expansion of their ranges to eastern South America and dispersal to habitats west of the Andes.

Other examples of the influence of the Andean uplift on speciation and range dynamics of *Prepona* clades are exemplified in the clade containing *P. pylene, P. eugenes, P. gnorima,* and *P. deiphile*. Recent phylogenetic reconstructions and morphological analyses of the tribe split the previously widespread *Prepona pylene* (sensu Lamas, 2004) into *P. pylene, P. eugenes,* and *P. gnorima*. Our dating suggests that *Prepona gnorima,* distributed in Central America and Chocó, split from the eastern South American *P. pylene* + *P. eugenes* at ~5 Ma. Furthermore, *Prepona deiphile*, although currently considered a widespread species, is paraphyletic in our phylogeny, supporting a split into a Central American and South American clade (Ortiz‐Acevedo et al., 2017; Turrent Carriles & García Díaz, 2019) that probably happened during the last pulse of Andean uplift.

Preponini genera exhibit contrasting biogeographical histories, suggesting that they might have evolved under different evolutionary pressures and scenarios. Although we found no evidence for color pattern shifts being important in diversification, different responses to major geological events suggest that exploring other possible ecological traits as correlates of diversification could be profitable. Differences in natural history, and in particular larval host plant relationships, potentially underlie the differing biogeographical patterns within Preponini, as demonstrated in the closely related Neotropical charaxine tribe Anaeini (Toussaint et al., 2019). Unfortunately, knowledge of host plants in Preponini is still rather incomplete, although, researchers, collectors, and enthusiasts are working together to fill this gap in knowledge.

## 5 CONCLUSIONS

This first investigation of color pattern evolution in Preponini posits new hypotheses for the observed shifts in diversification across the group. We found that, contrary to what might have been expected, changes in wing color did not influence diversification rates in this group. Both the formation of the Isthmus of Panama and the uplift of the Andes mountain range positively impacted the diversification of Preponini. Landscape reconfiguration and ecological opportunity created by the complex emergence of the connection between Central and South America and mountain building allowed dispersal, colonization, and divergence in newly available niches. Further studies should focus on the mechanisms that triggered the striking change in color that happened rapidly in the genus *Prepona*. In particular, *Prepona* species are potentially involved in mimicry rings with *Callicore* and *Asterope*, which also show a high richness in the Amazon region, with a number of species restricted to this area.

## CONFLICT OF INTEREST

We declare no conflict of interests.

## AUTHOR CONTRIBUTION

**Elena Ortiz‐Acevedo:** Conceptualization (lead); Data curation (lead); Formal analysis (equal); Funding acquisition (lead); Investigation (lead); Methodology (equal); Writing‐original draft (equal); Writing‐review & editing (equal). **Juan Pablo Gomez:** Conceptualization (supporting); Data curation (supporting); Formal analysis (equal); Investigation (supporting); Methodology (equal); Writing‐original draft (equal); Writing‐review & editing (supporting). **Marianne Espeland:** Methodology (supporting); Writing‐review & editing (supporting). **Emmanuel F. A. Toussaint:** Methodology (supporting); Writing‐review & editing (supporting). **Keith R Willmott:** Conceptualization (supporting); Funding acquisition (supporting); Investigation (supporting); Writing‐review & editing (supporting).

## Supporting information

FigS1‐12

TableS1‐16

AppendixS1

## Data Availability

Molecular data are available through the GenBank repository, and the morphological data and phylogenetic reconstruction are available in the Dryad repository https://datadryad.org/stash/dataset/doi:10.5061/dryad.7d7wm37sk).

## References

[ece36816-bib-0001] Abramoff, M. D. (2004). Image processing with ImageJ. Biophotonics International, 11, 36–42.

[ece36816-bib-0002] Alfaro, M. E. , Santini, F. , Brock, C. , Alamillo, H. , Dornburg, A. , Rabosky, D. L. , Carnevale, G. , & Harmon, L. J. (2009). Nine exceptional radiations plus high turnover explain species diversity in jawed vertebrates. Proceedings of the National Academy of Sciences USA, 106, 13410–13414. 10.1073/pnas.0811087106 PMC2715324 19633192

[ece36816-bib-0003] Antonelli, A. , Zizka, A. , Carvalho, F. A. , Scharn, R. , Bacon, C. D. , Silvestro, D. , & Condamine, F. L. (2018). Amazonia is the primary source of Neotropical biodiversity. Proceedings of the National Academy of Sciences, 115, 6034–6039. 10.1073/pnas.1713819115 PMC6003360 29760058

[ece36816-bib-0004] Axelrod, D. I. , Kalin Arroyo, M. T. , & Raven, P. H. (1991). Historical development of temperate vegetation in the Americas. Revista Chilena de Historia Natural, 64, 413–446.

[ece36816-bib-0005] Bacon, C. D. , Silvestro, D. , Jaramillo, C. , Smith, B. T. , Chakrabarty, P. , & Antonelli, A. (2015). Biological evidence supports an early and complex emergence of the Isthmus of Panama (vol 112, pg 6110, 2015). Proceedings of the National Academy of Sciences USA, 112, E3631 10.1073/pnas.1511204112 PMC4434730 25918375

[ece36816-bib-0006] Baele, G. , Li, W. L. S. , Drummond, A. J. , Suchard, M. A. , & Lemey, P. (2013). Accurate model selection of relaxed molecular clocks in bayesian phylogenetics. Molecular Biology and Evolution, 30, 239–243. 10.1093/molbev/mss243 23090976 PMC3548314

[ece36816-bib-0007] Berthier, S. (2005). Thermoregulation and spectral selectivity of the tropical butterfly *Prepona meander*: A remarkable example of temperature auto‐regulation. Applied Physics A, 80, 1397–1400. 10.1007/s00339-004-3185-x

[ece36816-bib-0008] Bouckaert, R. , Heled, J. , Kuhnert, D. , Vaughan, T. , Wu, C. H. , Xie, D. , Suchard, M. A. , Rambaut, A. , & Drummond, A. J. (2014). BEAST 2: A software platform for bayesian evolutionary analysis. PLoS Computational Biology, 10, 10.1371/journal.pcbi.1003537 PMC3985171 24722319

[ece36816-bib-0773] Bouckaert, R. R. , & Drummond, A. J. (2017). BModelTest: Bayesian Phylogenetic Site Model Averaging and Model Comparison. Bmc Evolutionary Biology 17 (February 6, 2017). 10.1186/s12862-017-0890-6 PMC5294809 28166715

[ece36816-bib-0009] Brakefield, P. M. , Gates, J. , Keys, D. , Kesbeke, F. , Wijngaarden, P. J. , Montelro, A. , French, V. , & Carroll, S. B. (1996). Development, plasticity and evolution of butterfly eyespot patterns. Nature, 384, 236 10.1038/384236a0 12809139

[ece36816-bib-0010] Buerki, S. , Forest, F. , Alvarez, N. , Nylander, J. A. A. , Arrigo, N. , & Sanmartin, I. (2011). An evaluation of new parsimony‐based versus parametric inference methods in biogeography: A case study using the globally distributed plant family Sapindaceae. Journal of Biogeography, 38, 531–550. 10.1111/j.1365-2699.2010.02432.x

[ece36816-bib-0011] Chazot, N. , Condamine, F. L. , Dudas, G. , Peña, C. , Matos‐Maraví, P. , Freitas, A. V. L. , Willmott, K. R. , Elias, M. , Warren, A. , Aduse‐Poku, K. , Lohman, D. J. , Penz, C. M. , DeVries, P. , Kodandaramaiah, U. , Fric, Z. F. , Nylin, S. , Müller, C. , Wheat, C. , Kawahara, A. Y. , … Wahlberg, N. (2020). The latitudinal diversity gradient in brush‐footed butterflies (Nymphalidae): Conserved ancestral tropical niche but different continental histories. bioRxiv 2020.04.16.045575. 10.1101/2020.04.16.045575 PMC8481491 34588433

[ece36816-bib-0012] Chazot, N. , Willmott, K. R. , Condamine, F. L. , De‐Silva, D. L. , Freitas, A. V. L. , Lamas, G. , Morlon, H. , Giraldo, C. E. , Jiggins, C. D. , Joron, M. , Mallet, J. , Uribe, S. , & Elias, M. (2016). Into the Andes: Multiple independent colonizations drive montane diversity in the Neotropical clearwing butterflies Godyridina. Molecular Ecology, 25, 5765–5784. 10.1111/mec.13773 27718282

[ece36816-bib-0013] Chazot, N. , Willmott, K. R. , Endara, P. G. S. , Toporov, A. , Hill, R. I. , Jiggins, C. D. , & Elias, M. (2014). Mutualistic mimicry and filtering by altitude shape the structure of andean butterfly communities. The American Naturalist, 183, 26–39. 10.1086/674100 24334733

[ece36816-bib-0014] Chazot, N. , Willmott, K. R. , Lamas, G. , Freitas, A. V. L. , Piron‐Prunier, F. , Arias, C. F. , Mallet, J. , De‐Silva, D. L. , & Elias, M. (2019). Renewed diversification following Miocene landscape turnover in a Neotropical butterfly radiation. Global Ecology and Biogeography, 28, 1118–1132. 10.1111/geb.12919

[ece36816-bib-0015] Clark, J. R. , Ree, R. H. , Alfaro, M. E. , King, M. G. , Wagner, W. L. , & Roalson, E. H. (2008). A comparative study in ancestral range reconstruction methods: Retracing the uncertain histories of insular lineages. Systematic Biology, 57, 693–707. 10.1080/10635150802426473 18853357

[ece36816-bib-0016] Condamine, F. L. , Silva‐Brandao, K. L. , Kergoat, G. J. , & Sperling, F. A. H. (2012). Biogeographic and diversification patterns of Neotropical Troidini butterflies (Papilionidae) support a museum model of diversity dynamics for Amazonia. BMC Evolutionary Biology, 12, 10.1186/1471-2148-12-82 PMC3464124 22690927

[ece36816-bib-0017] Darwin, C. (1874). The descent of man and selection in relation to sex, 2nd ed London.

[ece36816-bib-0018] Darwin, C. (1880). The sexual colors of certain butterflies. Nature, 21, 237.

[ece36816-bib-0019] Descimon, H. (1977). Biogéographie, mimétisme et spéciation dans le genre *Agrias* Doubleday (Lep. Nymphalidae Charaxinae). Publications Du Laboratoire De Zoologie De L’ecole Normale Supérieure, 9, 307–344.

[ece36816-bib-0020] De‐Silva, D. L. , Elias, M. , Willmott, K. , Mallet, J. , & Day, J. J. (2016). Diversification of clearwing butterflies with the rise of the Andes. Journal of Biogeography, 43, 44–58. 10.1111/jbi.12611 27546953 PMC4973677

[ece36816-bib-0021] De‐Silva, D. L. , Mota, L. L. , Chazot, N. , Mallarino, R. , Silva‐Brandao, K. L. , Pinerez, L. M. G. , Freitas, A. V. L. , Lamas, G. , Joron, M. , Mallet, J. , Giraldo, C. E. , Uribe, S. , Sarkinen, T. , Knapp, S. , Jiggins, C. D. , Willmott, K. R. , & Elias, M. (2017). North Andean origin and diversification of the largest ithomiine butterfly genus. Scientific Reports, 7, 1–17. 10.1038/srep45966 28387233 PMC5384087

[ece36816-bib-0022] Eastman, J. M. , Alfaro, M. E. , Joyce, P. , Hipp, A. L. , & Harmon, L. J. (2011). A novel comparative method for identifying shifts in the rate of character evolution on trees. Evolution, 65, 3578–3589. 10.1111/j.1558-5646.2011.01401.x 22133227

[ece36816-bib-0023] Espeland, M. , Breinholt, J. , Willmott, K. R. , Warren, A. D. , Vila, R. , Toussaint, E. F. A. , Maunsell, S. C. , Aduse‐Poku, K. , Talavera, G. , Eastwood, R. , Jarzyna, M. A. , Guralnick, R. , Lohman, D. J. , Pierce, N. E. , & Kawahara, A. Y. (2018). A comprehensive and dated phylogenomic analysis of butterflies. Current Biology, 28, 770–778. e5 10.1016/j.cub.2018.01.061 29456146

[ece36816-bib-0024] Fan, Y. , Wu, R. , Chen, M. H. , Kuo, L. , & Lewis, P. O. (2011). Choosing among partition models in Bayesian phylogenetics. Molecular Biology and Evolution, 28, 523–532. 10.1093/molbev/msq224 20801907 PMC3002242

[ece36816-bib-0025] Felsenstein, J. (2004). Inferring phylogenies. Sinauer.

[ece36816-bib-0026] Finkbeiner, S. D. , Briscoe, A. D. , & Reed, R. D. (2014). Warning signals are seductive: Relative contributions of color and pattern to predator avoidance and mate attraction in *Heliconius* butterflies. Evolution, 68, 3410–3420. 10.1111/evo.12524 25200939

[ece36816-bib-0027] FitzJohn, R. G. (2010). Quantitative traits and diversification. Systematic Biology, 59, 619–633. 10.1093/sysbio/syq053 20884813

[ece36816-bib-0028] Fordyce, J. A. , Nice, C. C. , Forister, M. L. , & Shapiro, A. M. (2002). The significance of wing pattern diversity in the Lycaenidae: Mate discrimination by two recently diverged species. Journal of Evolutionary Biology, 15, 871–879. 10.1046/j.1420-9101.2002.00432.x

[ece36816-bib-0029] Forest, F. (2009). Calibrating the Tree of Life: Fossils, molecules and evolutionary timescales. Annals of Botany, 104, 789–794. 10.1093/aob/mcp192 19666901 PMC2749537

[ece36816-bib-0030] Garzione, C. N. , Hoke, G. D. , Libarkin, J. C. , Withers, S. , MacFadden, B. , Eiler, J. , Ghosh, P. , & Mulch, A. (2008). Rise of the andes. Science, 320, 1304–1307. 10.1126/science.1148615 18535236

[ece36816-bib-0031] Harmon, L. J. , Weir, J. T. , Brock, C. D. , Glor, R. E. , & Challenger, W. (2008). GEIGER: Investigating evolutionary radiations. Bioinformatics, 24, 129–131. 10.1093/bioinformatics/btm538 18006550

[ece36816-bib-0032] Harvey, M. G. , & Rabosky, D. L. (2018). Continuous traits and speciation rates: Alternatives to state‐dependent diversification models. Methods in Ecology and Evolution, 9, 984–993. 10.1111/2041-210X.12949

[ece36816-bib-0033] Hoorn, C. , Wesselingh, F. P. , ter Steege, H. , Bermudez, M. A. , Mora, A. , Sevink, J. , Sanmartin, I. , Sanchez‐Meseguer, A. , Anderson, C. L. , Figueiredo, J. P. , Jaramillo, C. , Riff, D. , Negri, F. R. , Hooghiemstra, H. , Lundberg, J. , Stadler, T. , Sarkinen, T. , & Antonelli, A. (2010). Amazonia through time: Andean uplift, climate change, landscape evolution, and biodiversity. Science, 330, 927–931. 10.1126/science.1194585 21071659

[ece36816-bib-0034] Jenkins, D. W. (1987). Neotropical Nymphalidae VI. Revision of Asterope (=Callithea Auct.). Bulletin of the Allyn Museum, 1–66.

[ece36816-bib-0035] Jiggins, C. D. (2008). Ecological speciation in mimetic butterflies. BioScience, 58, 541–548. 10.1641/B580610

[ece36816-bib-0036] Jiggins, C. D. , Mallarino, R. , Willmott, K. R. , & Bermingham, E. (2006). The phylogenetic pattern of speciation and wing pattern change in neotropical *Ithomia* butterflies (Lepidoptera: Nymphalidae). Evolution, 60, 1454–1466. 16929662 10.1554/05-483.1

[ece36816-bib-0037] Jiggins, C. , Naisbit, R. , Coe, R. , & Mallet, J. (2001). Reproductive isolation caused by color pattern mimicry. Nature, 411, 302–305. 11357131 10.1038/35077075

[ece36816-bib-0038] Josse, C. , Navarro, G. , Comer, P. , Evans, R. , Faber‐Langendoen, D. , Fellows, M. , Kittel, G. , Menard, S. , Pyne, M. , Reid, M. , Schulz, K. , Snow, K. & , & Teague, J. (2003). Ecological systems of Latin America and the Caribbean: A working classification of terrestrial systems, Arlington, VA: NatureServe.

[ece36816-bib-0039] Kemp, D. J. (2007). Female butterflies prefer males bearing bright iridescent ornamentation. Proceedings of the Royal Society B: Biological Sciences, 274, 1043–1047. 10.1098/rspb.2006.0043 PMC2124467 17284412

[ece36816-bib-0040] Kürschner, W. M. , Kvaček, Z. , & Dilcher, D. L. (2008). The impact of Miocene atmospheric carbon dioxide fluctuations on climate and the evolution of terrestrial ecosystems. Proceedings of the National Academy of Sciences, 105, 449–453. 10.1073/pnas.0708588105 PMC2206556 18174330

[ece36816-bib-0041] Lagomarsino, L. P. , Condamine, F. L. , Antonelli, A. , Mulch, A. , & Davis, C. C. (2016). The abiotic and biotic drivers of rapid diversification in Andean bellflowers (Campanulaceae). New Phytologist, 210, 1430–1442. 10.1111/nph.13920 26990796 PMC4950005

[ece36816-bib-0042] Lamas, G. (2004). Checklist: Part 4A. Hesperioidea – Papilionoidea In Heppner J. B. (Ed.), Atlas of Neotropical Lepidoptera (p. 439). Association for Tropical Lepidoptera and Scientific Publishers.

[ece36816-bib-0043] Lanfear, R. , Calcott, B. , Ho, S. Y. W. , & Guindon, S. (2012). PartitionFinder: Combined selection of partitioning schemes and substitution models for phylogenetic analyses. Molecular Biology and Evolution, 29, 1695–1701. 10.1093/molbev/mss020 22319168

[ece36816-bib-0044] Losos, J. B. , Warheit, K. I. , & Schoener, T. W. (1997). Adaptive differentiation following experimental island colonization in *Anolis* lizards. Nature, 387, 70–73. 10.1038/387070a0

[ece36816-bib-0045] Louca, S. , & Doebeli, M. (2017). Efficient comparative phylogenetics on large trees. Bioinformatics, 34, 1053–1055. 10.1093/bioinformatics/btx701 29091997

[ece36816-bib-0046] Louca, S. , & Pennell, M. W. (2020). Extant time trees are consistent with a myriad of diversification histories. Nature, 580, 502–505. 10.1038/s41586-020-2176-1 32322065

[ece36816-bib-0047] Magallon, S. , & Sanderson, M. J. (2001). Absolute diversification rates in angiosperm clades. Evolution, 55, 1762–1780. 10.1111/j.0014-3820.2001.tb00826.x 11681732

[ece36816-bib-0048] Mallet, J. , & Gilbert, L. E. (1995). Why are there so many mimicry rings? Correlations between habitat, behaviour and mimicry in *Heliconius* butterflies. Biological Journal of the Linnean Society, 55, 159–180. 10.1111/j.1095-8312.1995.tb01057.x

[ece36816-bib-0049] Matzke, N. J. (2014). Model selection in historical biogeography reveals that founder‐event speciation is a crucial process in island clades. Systematic Biology, 64, 167 10.1093/sysbio/syu091 25123369

[ece36816-bib-0050] Matzke, N. J. (2018). BioGeoBEARS: BioGeography with Bayesian (and likelihood) evolutionary analysis with R scripts. GitHub. 10.5281/zenodo.1478250

[ece36816-bib-0051] Mavarez, J. , Salazar, C. A. , Bermingham, E. , Salcedo, C. , Jiggins, C. D. , & Linares, M. (2006). Speciation by hybridization in *Heliconius* butterflies. Nature, 441, 868–871. 10.1038/nature04738 16778888

[ece36816-bib-0052] Mayhew, P. J. (2007). Why are there so many insect species? Perspectives from fossils and phylogenies. Biological Reviews of the Cambridge Philosophical Society, 82, 425–454. 10.1111/j.1469-185X.2007.00018.x 17624962

[ece36816-bib-0053] Montes, C. , Cardona, A. , Jaramillo, C. , Pardo, A. , Silva, J. C. , Valencia, V. , Ayala, C. , Perez‐Angel, L. C. , Rodriguez‐Parra, L. A. , Ramirez, V. , & Nino, H. (2015). Middle Miocene closure of the Central American Seaway. Science, 348, 226–229. 10.1126/science.aaa2815 25859042

[ece36816-bib-0054] Morlon, H. (2014). Phylogenetic approaches for studying diversification. Ecology Letters, 17, 508–525. 10.1111/ele.12251 24533923

[ece36816-bib-0055] Morlon, H. , Hartig, F. , & Robin, S. (2020). Prior hypotheses or regularization allow inference of diversification histories from extant timetrees. bioRxiv. 2020.07.03.185074. 10.1101/2020.07.03.185074

[ece36816-bib-0056] Morlon, H. , Lewitus, E. , Condamine, F. L. , Manceau, M. , Clavel, J. , & Drury, J. (2016). RPANDA: An R package for macroevolutionary analyses on phylogenetic trees. Methods in Ecology and Evolution, 7, 589–597. 10.1111/2041-210X.12526

[ece36816-bib-0057] Morlon, H. , Parsons, T. L. , & Plotkin, J. B. (2011). Reconciling molecular phylogenies with the fossil record. Proceedings of the National Academy of Sciences, 108, 16327–16332. 10.1073/pnas.1102543108 PMC3182748 21930899

[ece36816-bib-0058] Mullen, S. P. , Savage, W. K. , Wahlberg, N. , & Willmott, K. R. (2011). Rapid diversification and not clade age explains high diversity in neotropical *Adelpha* butterflies. Proceedings of the Royal Society B: Biological Sciences, 278, 1777–1785. 10.1098/rspb.2010.2140 PMC3097834 21106589

[ece36816-bib-0059] Nadeau, N. J. , Pardo‐Diaz, C. , Whibley, A. , Supple, M. A. , Saenko, S. V. , Wallbank, R. W. R. , Wu, G. C. , Maroja, L. , Ferguson, L. , Hanly, J. J. , Hines, H. , Salazar, C. , Merrill, R. M. , Dowling, A. J. , ffrench‐Constant, R. H. , Llaurens, V. , Joron, M. , McMillan, W. O. , & Jiggins, C. D. (2016). The gene cortex controls mimicry and crypsis in butterflies and moths. Nature, 534, 106–110. 10.1038/nature17961 27251285 PMC5094491

[ece36816-bib-0060] Neild, A. F. E. (1996). The Butterflies of Venezuela. Part 1: Nymphalidae I (Limenitidinae, Apaturinae, Charaxinae). A comprehensive guide to the identification of adult Nymphalidae, Papilionidae, and Pieridae. Meridian Publications.

[ece36816-bib-0061] Nicholson, D. B. , Ross, A. J. , & Mayhew, P. J. (2014). Fossil evidence for key innovations in the evolution of insect diversity. Proceedings of the Royal Society B: Biological Sciences, 281, 1–7. 10.1098/rspb.2014.1823 PMC4173696 25165766

[ece36816-bib-0062] Nijhout, F. (1991). The development and evolution of the butterfly wing patterns. Smithsonian Institution Press.

[ece36816-bib-0063] Obara, Y. , & Majerus, M. E. N. (2000). Initial Mate Recognition in the British Cabbage Butterfly, Pieris rapae rapae. Zoological Science, 17, 725–730. 10.2108/zsj.17.725

[ece36816-bib-0064] Oliver, J. C. , Tong, X.‐L. , Gall, L. F. , Piel, W. H. , & Monteiro, A. (2012). A single origin for nymphalid butterfly eyespots followed by widespread loss of associated gene expression. PLOS Genetics, 8, e1002893 10.1371/journal.pgen.1002893 22916033 PMC3420954

[ece36816-bib-0065] Ortiz‐Acevedo, E. , Bonfantti, D. , Casagrande, M. , Mielke, O. H. H. , Espeland, M. , & Willmott, K. R. (2017). Using molecules and morphology to unravel the systematics of neotropical preponine butterflies (Lepidoptera: Charaxinae: Preponini). Insect Systematics and Diversity, 1, 48–56. 10.1093/isd/ixx002

[ece36816-bib-0066] Ortiz‐Acevedo, E. , & Willmott, K. R. (2013). Molecular systematics of the butterfly tribe Preponini (Nymphalidae: Charaxinae). Systematic Entomology, 38, 440–449. 10.1111/syen.12008

[ece36816-bib-0067] Paradis, E. , Claude, J. , & Strimmer, K. (2004). APE: Analyses of phylogenetics and evolution in R language. Bioinformatics, 20, 289–290. 10.1093/bioinformatics/btg412 14734327

[ece36816-bib-0068] Peña, C. , & Espeland, M. (2015). Diversity dynamics in nymphalidae butterflies: Effect of phylogenetic uncertainty on diversification rate shift estimates. PLoS One, 10, e0120928 10.1371/journal.pone.0120928 25830910 PMC4382342

[ece36816-bib-0069] Peña, C. , & Wahlberg, N. (2008). Prehistorical climate change increased diversification of a group of butterflies. Biology Letters, 4, 274–278. 10.1098/rsbl.2008.0062 18364308 PMC2610051

[ece36816-bib-0070] Pinheiro, C. E. G. , & Freitas, A. V. L. (2014). Some possible cases of escape mimicry in neotropical butterflies. Neotropical Entomology, 43, 393–398. 10.1007/s13744-014-0240-y 27193948

[ece36816-bib-0071] Pinto‐Sanchez, N. R. , Ibanez, R. , Madrinan, S. , Sanjur, O. I. , Bermingham, E. , & Crawford, A. J. (2012). The Great American Biotic Interchange in frogs: Multiple and early colonization of Central America by the South American genus *Pristimantis* (Anura: Craugastoridae). Molecular Phylogenetics and Evolution, 62, 954–972. 10.1016/j.ympev.2011.11.022 22178362

[ece36816-bib-0072] Pound, M. J. , Haywood, A. M. , Salzmann, U. , Riding, J. B. , Lunt, D. J. , & Hunter, S. J. (2011). A Tortonian (Late Miocene, 11.61–7.25Ma) global vegetation reconstruction. Palaeogeography, Palaeoclimatology, Palaeoecology, 300, 29–45. 10.1016/j.palaeo.2010.11.029

[ece36816-bib-0073] Pybus, O. G. , & Harvey, P. H. (2000). Testing macro‐evolutionary models using incomplete molecular phylogenies. Proceedings: Biological Sciences, 267, 2267–2272. 10.1098/rspb.2000.1278 11413642 PMC1690817

[ece36816-bib-0074] Rabosky, D. L. (2014). Automatic detection of key innovations, rate shifts, and diversity‐dependence on phylogenetic trees. PLoS One, 9, e89543 10.1371/journal.pone.0089543 24586858 PMC3935878

[ece36816-bib-0075] Raftery, A. E. , Satagopan, J. M. , Newton, M. A. , & Krivitsky, P. N. (2007). Estimating the integrated likelihood via posterior simulation using the harmonic mean identity In Bernardo J. M., Bayarri M. J., Berger J. O., Dawid A. P., Heckerman D., Smith A. F. M., & West M. (Eds.), Bayesian statistics (pp. 1–45). Oxford University Press.

[ece36816-bib-0076] Rahbek, C. , Borregaard, M. K. , Antonelli, A. , Colwell, R. K. , Holt, B. G. , Nogues‐Bravo, D. , Rasmussen, C. M. Ø. , Richardson, K. , Rosing, M. T. , Whittaker, R. J. , & Fjeldså, J. (2019). Building mountain biodiversity: Geological and evolutionary processes. Science, 365, 1114–1119. 10.1126/science.aax0151 31515384

[ece36816-bib-0077] Rambaut, A. , Suchard, M. A. , Xie, D. , & Drummond, A. (2014). Tracer v1.6.

[ece36816-bib-0078] Rasband, W. S. (1997). ImageJ. National Institute of Health.

[ece36816-bib-0079] Ree, R. H. , & Sanmartín, I. (2018). Conceptual and statistical problems with the DEC+J model of founder‐event speciation and its comparison with DEC via model selection. Journal of Biogeography, 45, 741–749. 10.1111/jbi.13173

[ece36816-bib-0080] Ree, R. H. , & Smith, S. A. (2008). Maximum likelihood inference of geographic range evolution by dispersal, local extinction, and cladogenesis. Systematic Biology, 57, 4–14. 10.1080/10635150701883881 18253896

[ece36816-bib-0081] Reed, R. D. , Papa, R. , Martin, A. , Hines, H. M. , Counterman, B. A. , Pardo‐Diaz, C. , Jiggins, C. D. , Chamberlain, N. L. , Kronforst, M. R. , Chen, R. , Halder, G. , Nijhout, H. F. , & McMillan, W. O. (2011). Optix drives the repeated convergent evolution of butterfly wing pattern mimicry. Science, 333, 1137–1141. 10.1126/science.1208227 21778360

[ece36816-bib-0082] Ronquist, F. (1997). Dispersal‐vicariance analysis: A new approach to the quantification of historical biogeography. Systematic Biology, 46, 195–203. 10.2307/2413643

[ece36816-bib-0083] Ronquist, F. , & Sanmartin, I. (2011). Phylogenetic methods in biogeography. Annual Review of Ecology, Evolution, and Systematics, 42, 441–464. 10.1146/annurev-ecolsys-102209-144710

[ece36816-bib-0084] Ruxton, G. D. , Speed, M. , & Sherratt, T. N. (2004). Evasive mimicry: When (if ever) could mimicry based on difficulty of capture evolve? Proceedings of the Royal Society B: Biological Sciences, 271, 2135–2142. 10.1098/rspb.2004.2816 PMC1691841 15475333

[ece36816-bib-0085] Sahoo, R. K. , Warren, A. D. , Collins, S. C. , & Kodandaramaiah, U. (2017). Hostplant change and paleoclimatic events explain diversification shifts in skipper butterflies (Family: Hesperiidae). BMC Evolutionary Biology, 17, 10.1186/s12862-017-1016-x PMC5541431 28768477

[ece36816-bib-0086] Schluter, D. (2000). The ecology of adaptive radiation. Oxford University Press.

[ece36816-bib-0087] Schneider, C. , Rasband, W. , & Eliceiri, K. (2012). NIH Image to ImageJ: 25 years of image analysis. Nature Methods, 9(7), 671–675. 10.1038/nmeth.2089 22930834 PMC5554542

[ece36816-bib-0088] Smith, B. T. , McCormack, J. E. , Cuervo, A. M. , Hickerson, M. J. , Aleixo, A. , Cadena, C. D. , Perez‐Eman, J. , Burney, C. W. , Xie, X. , Harvey, M. G. , Faircloth, B. C. , Glenn, T. C. , Derryberry, E. P. , Prejean, J. , Fields, S. , & Brumfield, R. T. (2014). The drivers of tropical speciation. Nature, 515, 406–409. 10.1038/nature13687 25209666

[ece36816-bib-0089] Strömberg, C. A. E. , Dunn, R. E. , Madden, R. H. , Kohn, M. J. , & Carlini, A. A. (2013). Decoupling the spread of grasslands from the evolution of grazer‐type herbivores in South America. Nature Communications, 4, 1478 10.1038/ncomms2508 23403579

[ece36816-bib-0090] Taper, M. L. , & Ponciano, J. M. (2016). Evidential statistics as a statistical modern synthesis to support 21st century science. Population Ecology, 58, 9–29. 10.1007/s10144-015-0533-y

[ece36816-bib-0091] Toussaint, E. F. A. , Dias, F. M. S. , Mielke, O. H. H. , Casagrande, M. M. , Sañudo‐Restrepo, C. P. , Lam, A. , Morinière, J. , Balke, M. , & Vila, R. (2019). Flight over the Proto‐Caribbean seaway: Phylogeny and macroevolution of Neotropical Anaeini leafwing butterflies. Molecular Phylogenetics and Evolution, 137, 86–103. 10.1016/j.ympev.2019.04.020 31022515

[ece36816-bib-0092] Turrent Carriles, A. , & García Días, J. J (2019). Descripción de una nueva especie de Prepona Boisduval, 1836 (Lepidoptera: Nymphalidae) de la Sierra Madre del Sur. Revista de la Sociedad Mexicana de Lepidopterología VII.

[ece36816-bib-0093] van Someren, V. G. L. , & Jackson, T. H. E. (1959). Some comments on protective resemblance amongst African Lepidoptera (Rhopalocera). Journal of the Lepidopterists’ Society, 13, 121–150.

[ece36816-bib-0094] Wahlberg, N. , Leneveu, J. , Kodandaramaiah, U. , Peña, C. , Nylin, S. , Freitas, A. V. L. , & Brower, A. V. Z. (2009). Nymphalid butterflies diversify following near demise at the Cretaceous/Tertiary boundary. Proceedings of the Royal Society B: Biological Sciences, 276, 4295–4302. 10.1098/rspb.2009.1303 PMC2817107 19793750

[ece36816-bib-0095] Wallace, A. R. (1877). The colours of animals and plants. The American Naturalist, 11, 713–728.

[ece36816-bib-0096] Wallace, A. R. (1889). Darwinism: An exposition of the theory of natural selection with some of its applications. Macmillan and Company.

[ece36816-bib-0097] Weir, J. T. , Bermingham, E. , & Schluter, D. (2009). The Great American Biotic Interchange in birds. Proceedings of the National Academy of Sciences USA, 106, 21737–21742. 10.1073/pnas.0903811106 PMC2799814 19996168

