# Supplementary material for: The roles of wing color pattern and geography in the evolution of Neotropical Preponini butterflies
Source: Ecol Evol. 2020 Oct 3;10(23):12801–16. doi: 10.1002/ece3.6816 (PMC7713932; doi:10.1002/ece3.6816)
Supplement: Supplementary file 1 — FigS1‐12 [file ECE3-10-12801-s001.docx]

**Supplementary Figures**

***
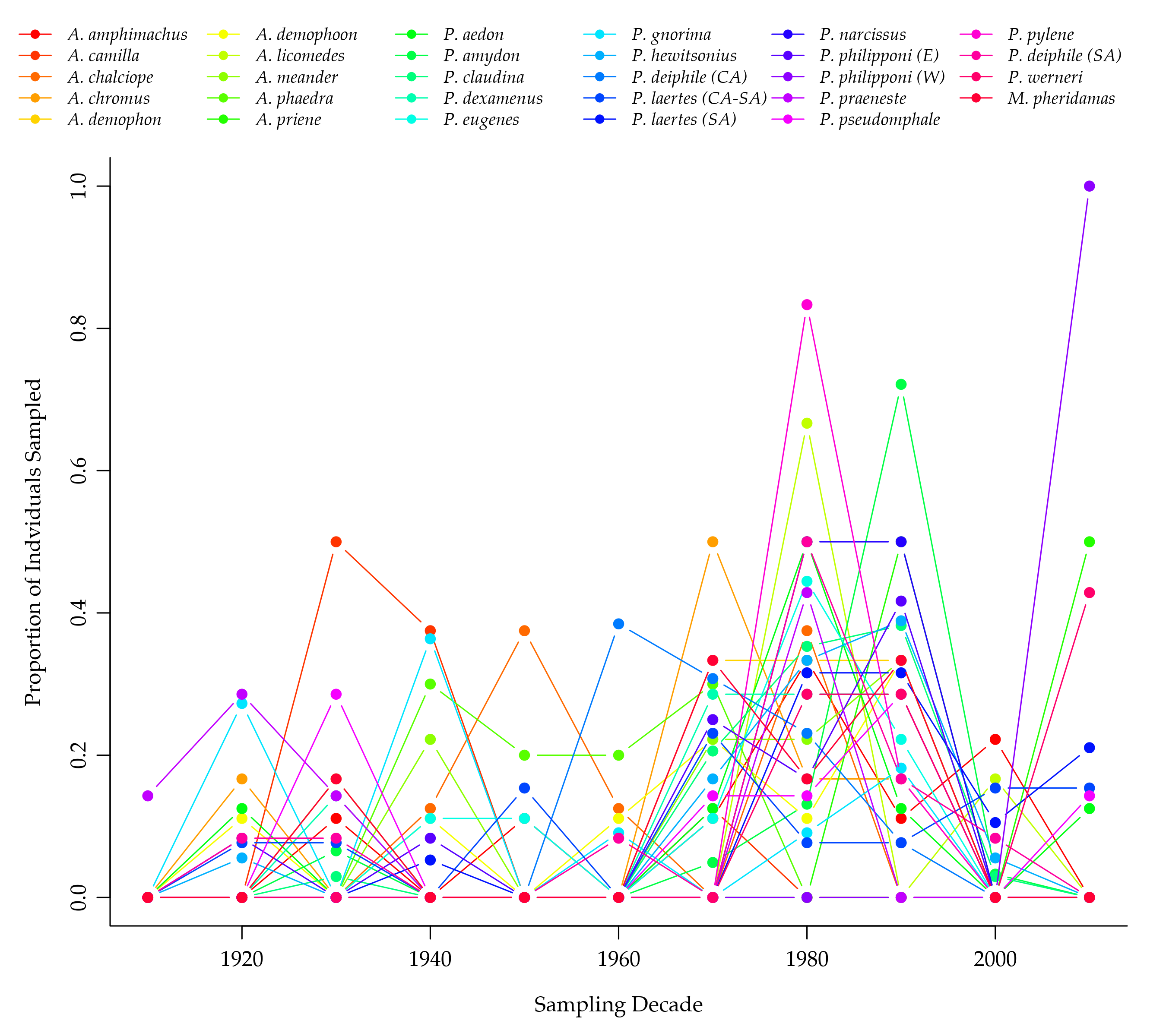
***

Figure S1. Collection dates for the specimens measured in the study of color.

***
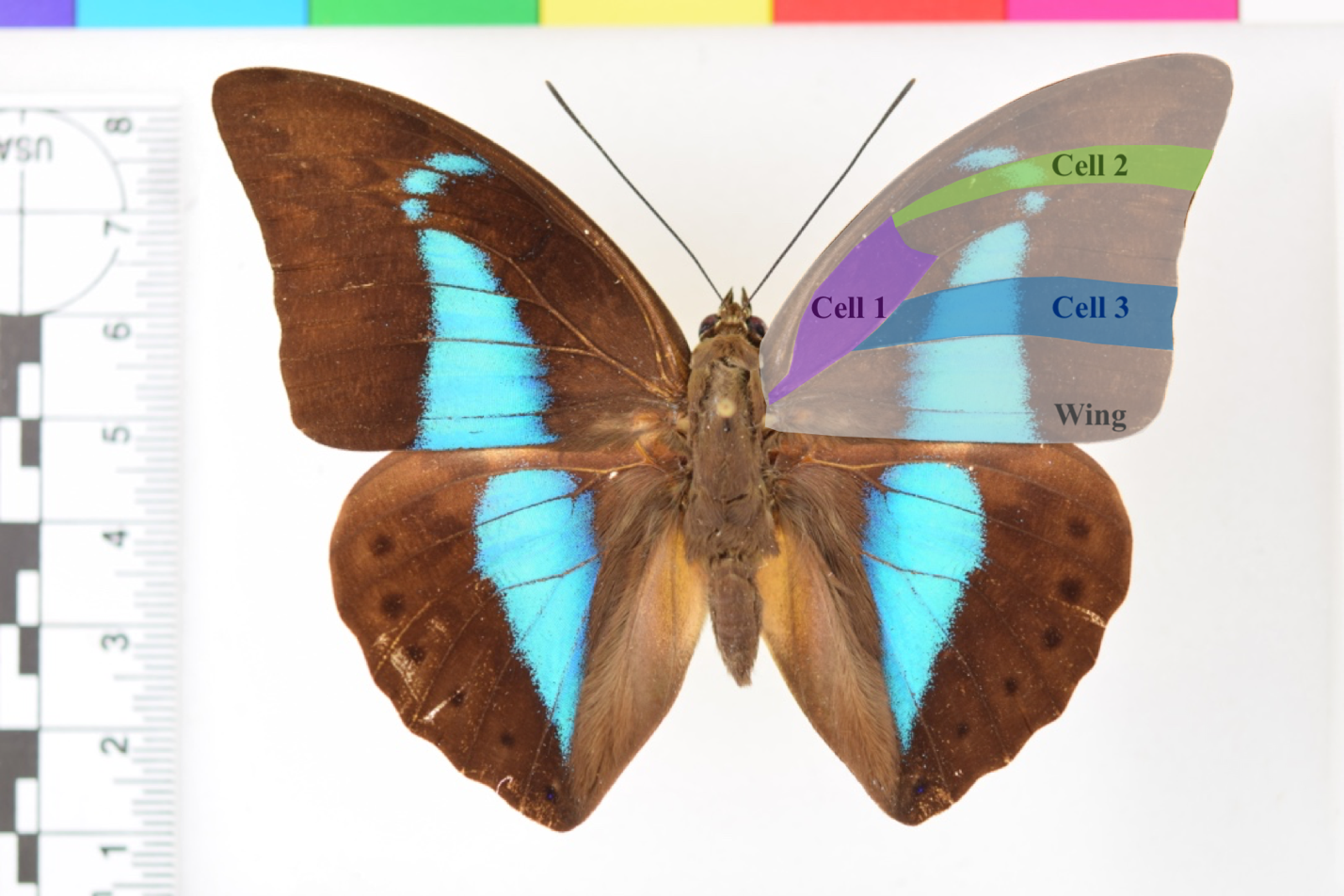
***

Figure S2. Wing regions measured in the study of color. Photo by EOA.


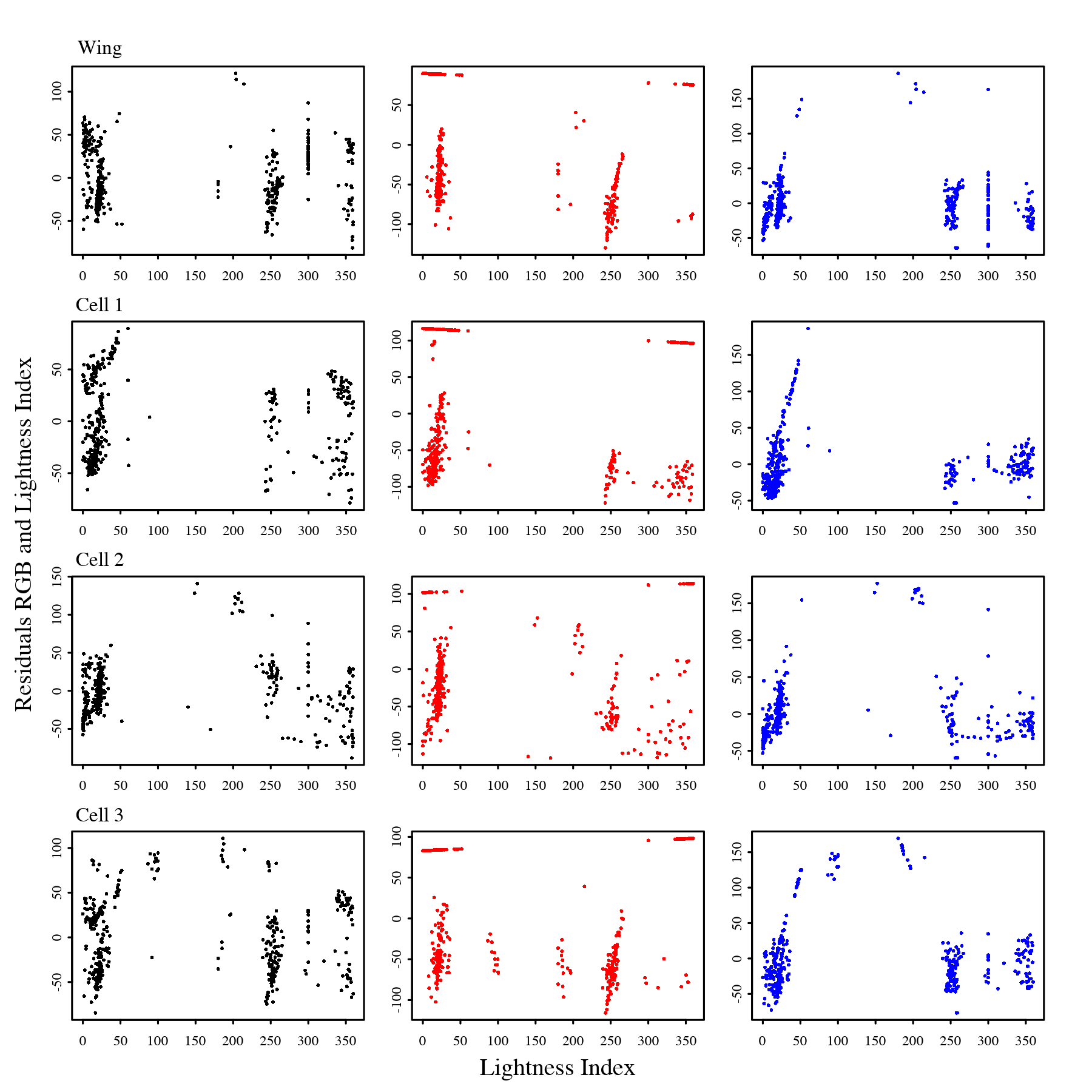


Figure S3. Residuals of lightness and RGB relationship plotted against lightness for each individual in the database showing no relationship between lightness and such residuals. Panels with black dots correspond R+G+B measurement, red dots correspond to the red channel measurement and blue dots correspond to the blue channel measurements.


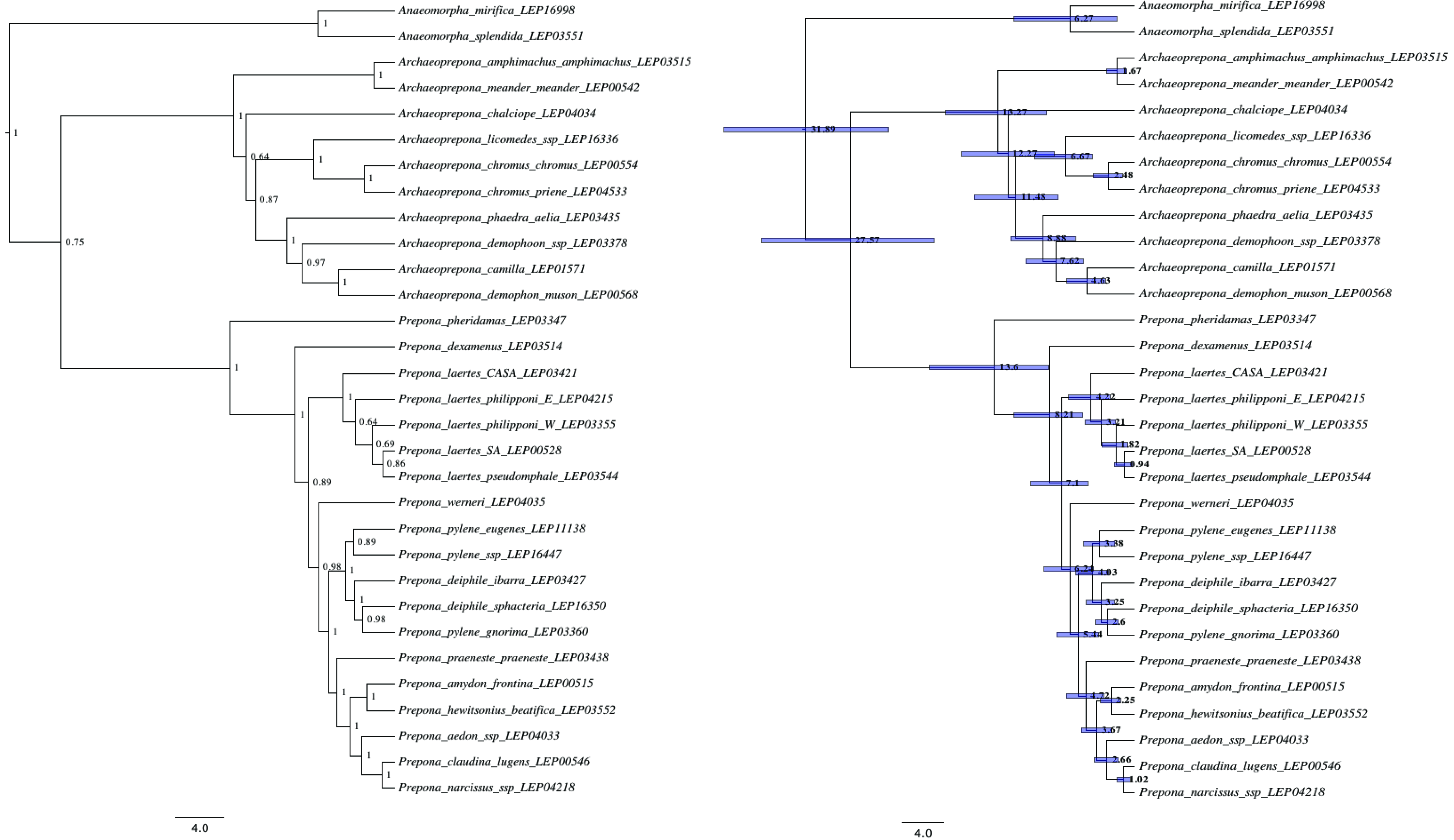


Figure S4. Preponini phylogeny showing posterior probability support (left) and dating credibility intervals (right).


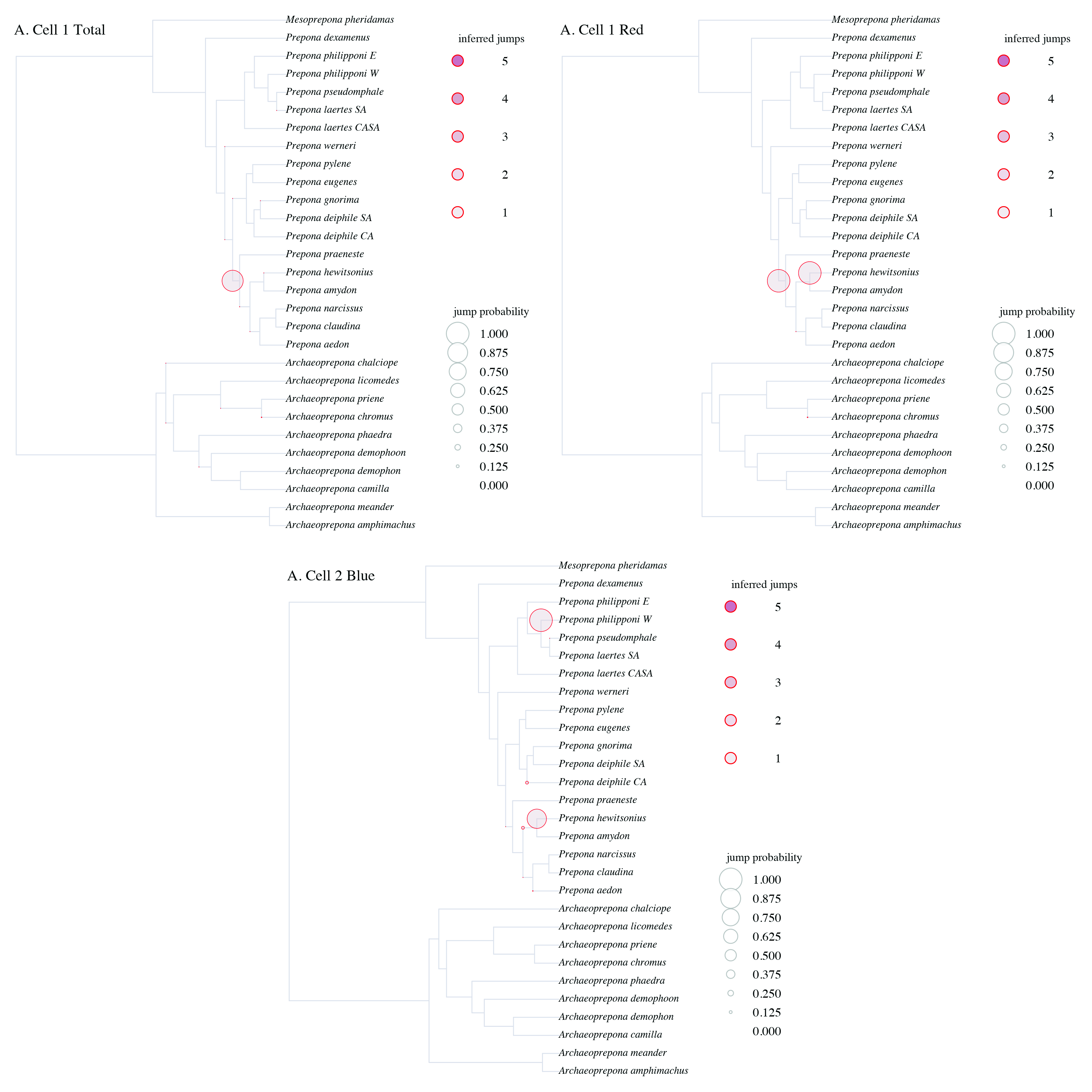


Figure S5. Inferred jumps for mode color measurements using the RGB raw data.


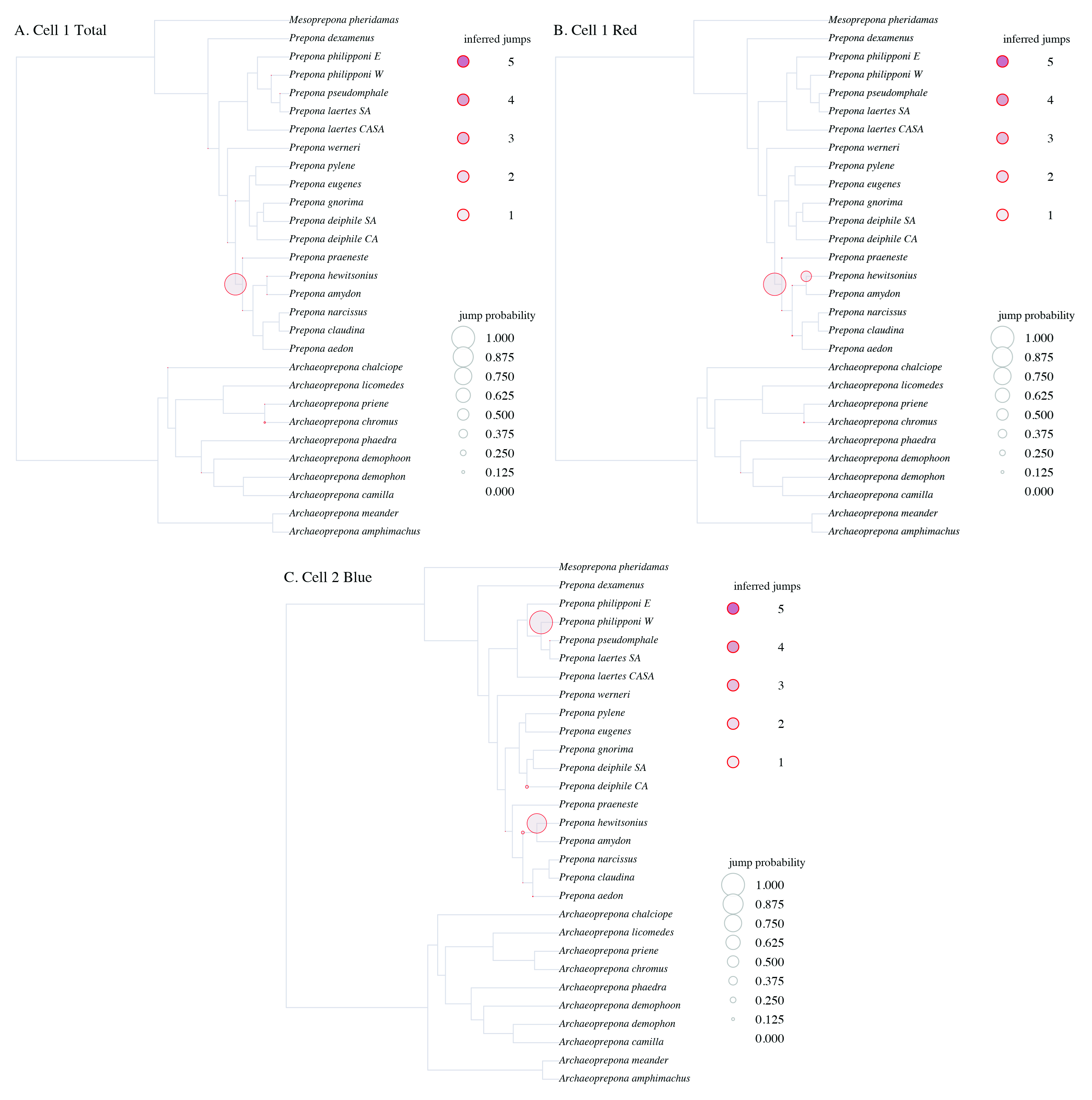


Figure S6. Inferred jumps for mode color measurements using the residuals of the relationship between RGB and lightness.

***
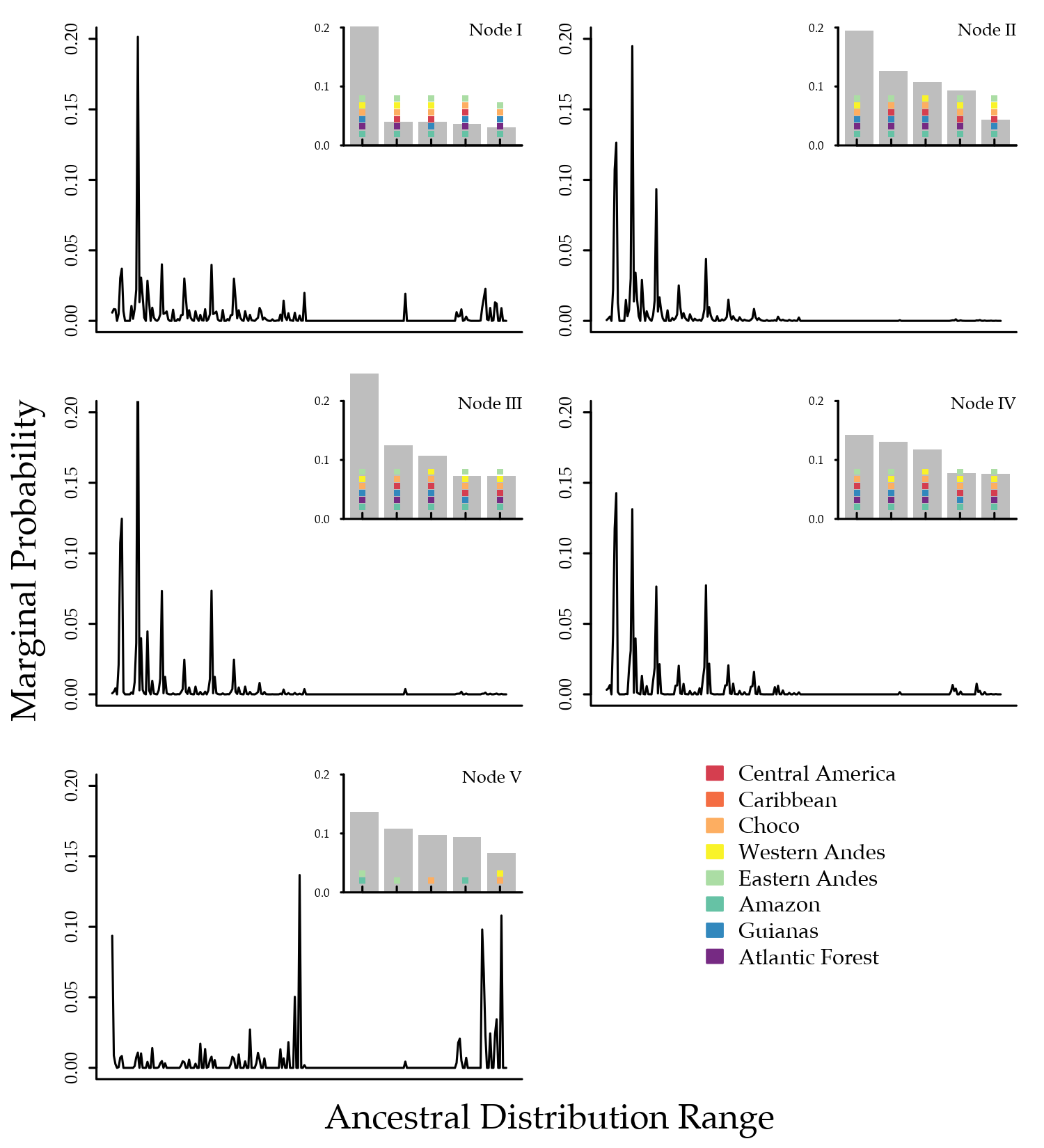
***

Figure S7. Marginal probability for all possible ranges of origin of the biogeographical estimation. The x axis corresponds to all the possible area combinations. The nodes correspond to the ones labeled in Figure 3.


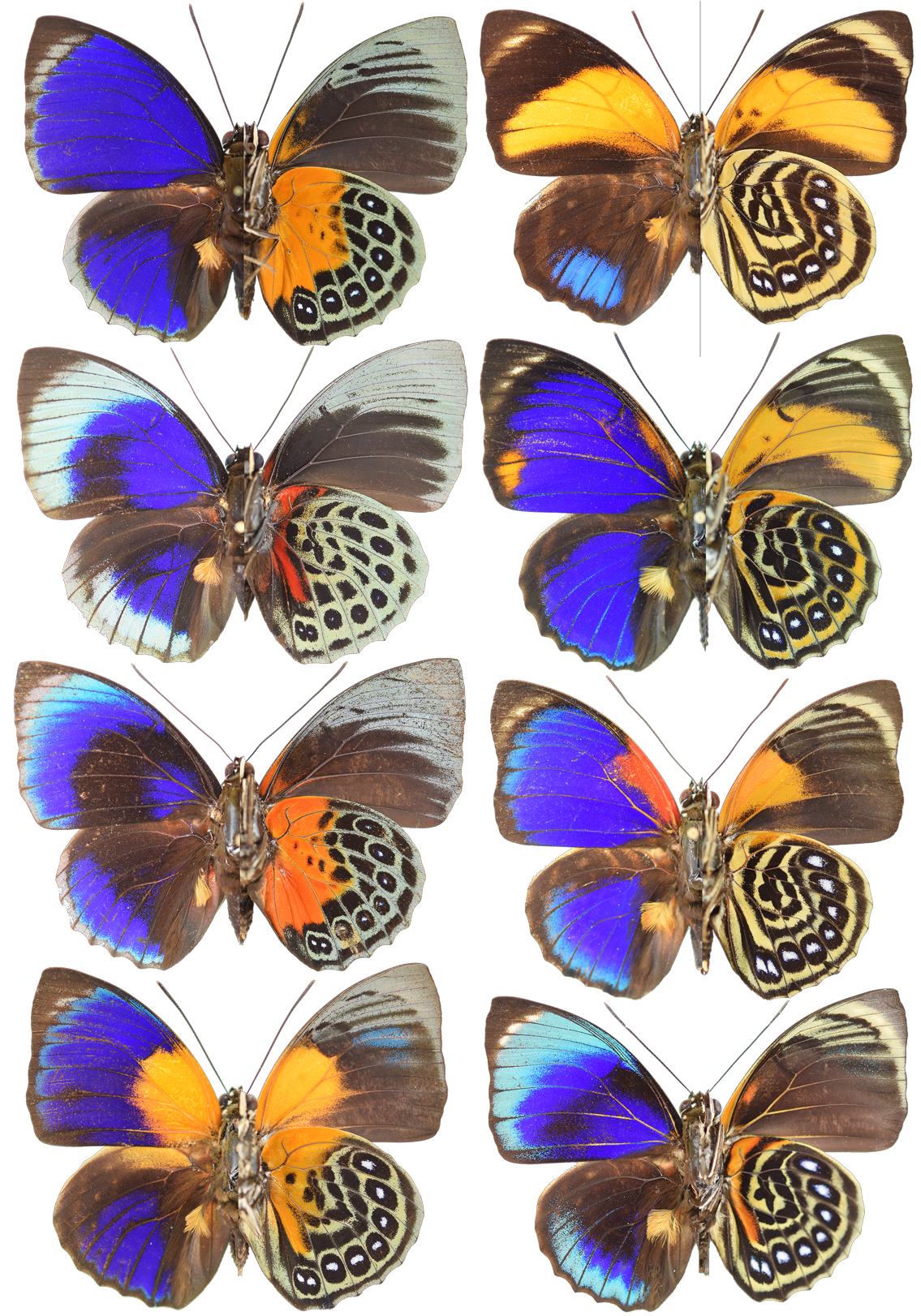


Figure S8. High phenotypic diversity in *Prepona* *hewitsonius* and *P. amydon*. Butterflies in left column correspond to (top to bottom): *Prepona* *hewitsonius* *stuarti*, *P. h. beata, P. h. beatifica* and *P. h. hewitsonius.* Butterflies in right column correspond to (top to bottom): *Prepona amydon boliviensis, P. a. excelsior, P. a. phalcidon* form 1 and *P. a.* *phalcidon* form 2. Photos by EOA.

***
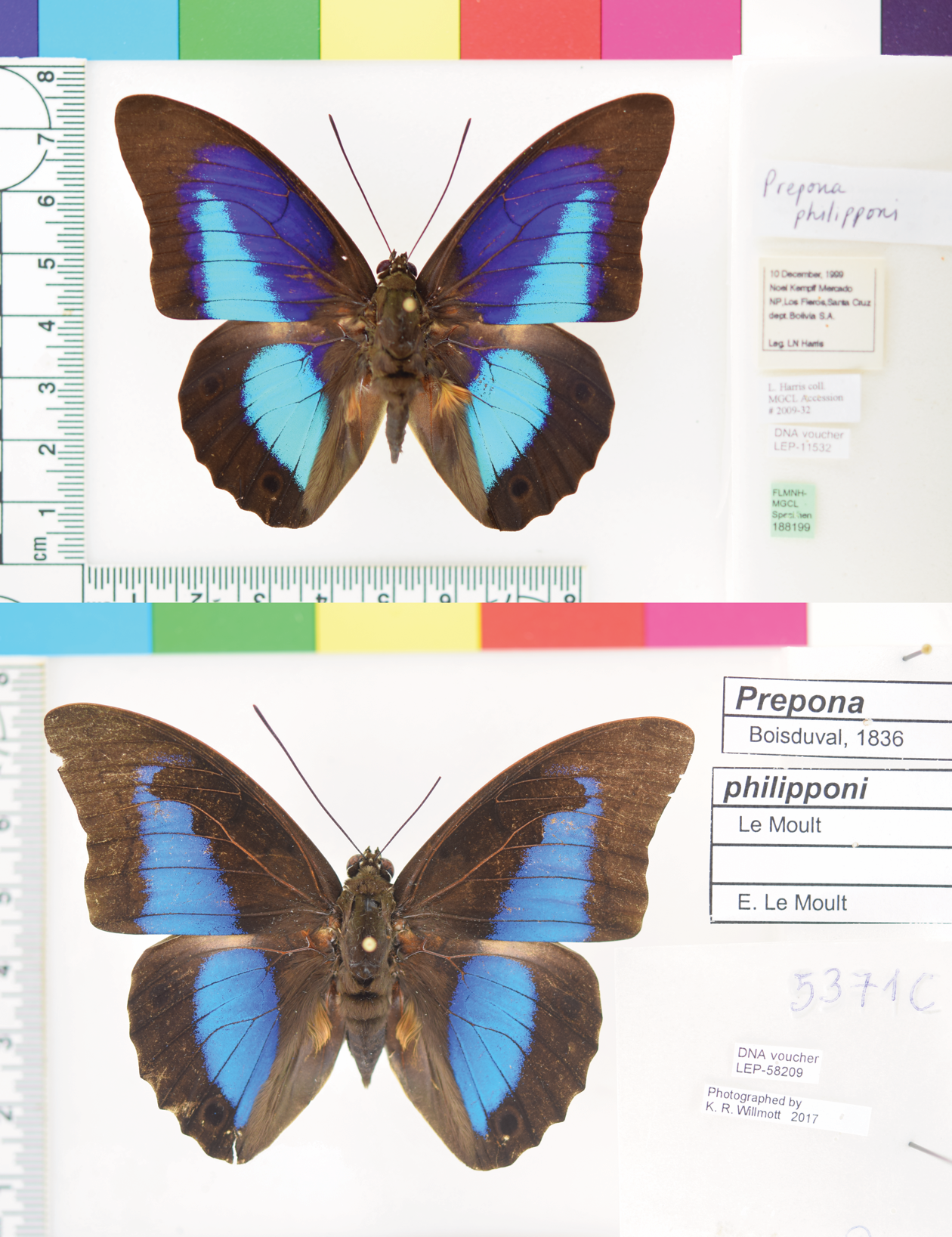
***

Figure S9. *Prepona philipponi (E)* (top) and *Prepona philipponi (W)* (bottom) dorsal coloration pattern. Photos by EOA.


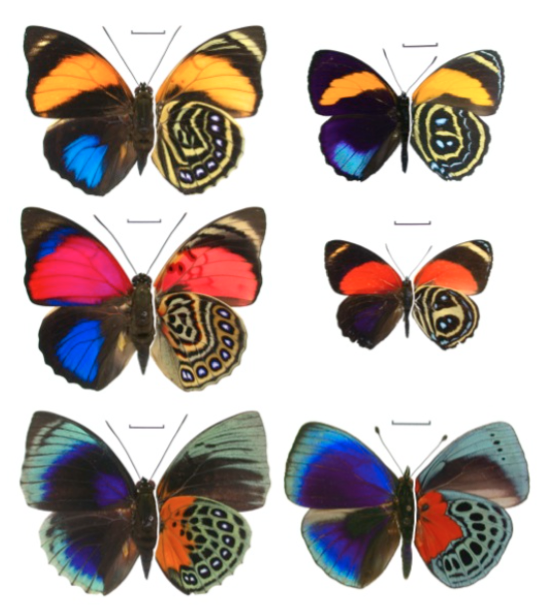


Figure S10. Potential involvement of *Prepona, Callicore*, and *Asterope* in mimicry rings. Butterflies shown (from top left to bottom right): *Prepona amydon zenodorus, Callicore excelsior pastazza, Prepona claudina, Callicore texa maimuna, Prepona hewitsonius,* and *Asterope leprieuri* Photos by: KRW.

***
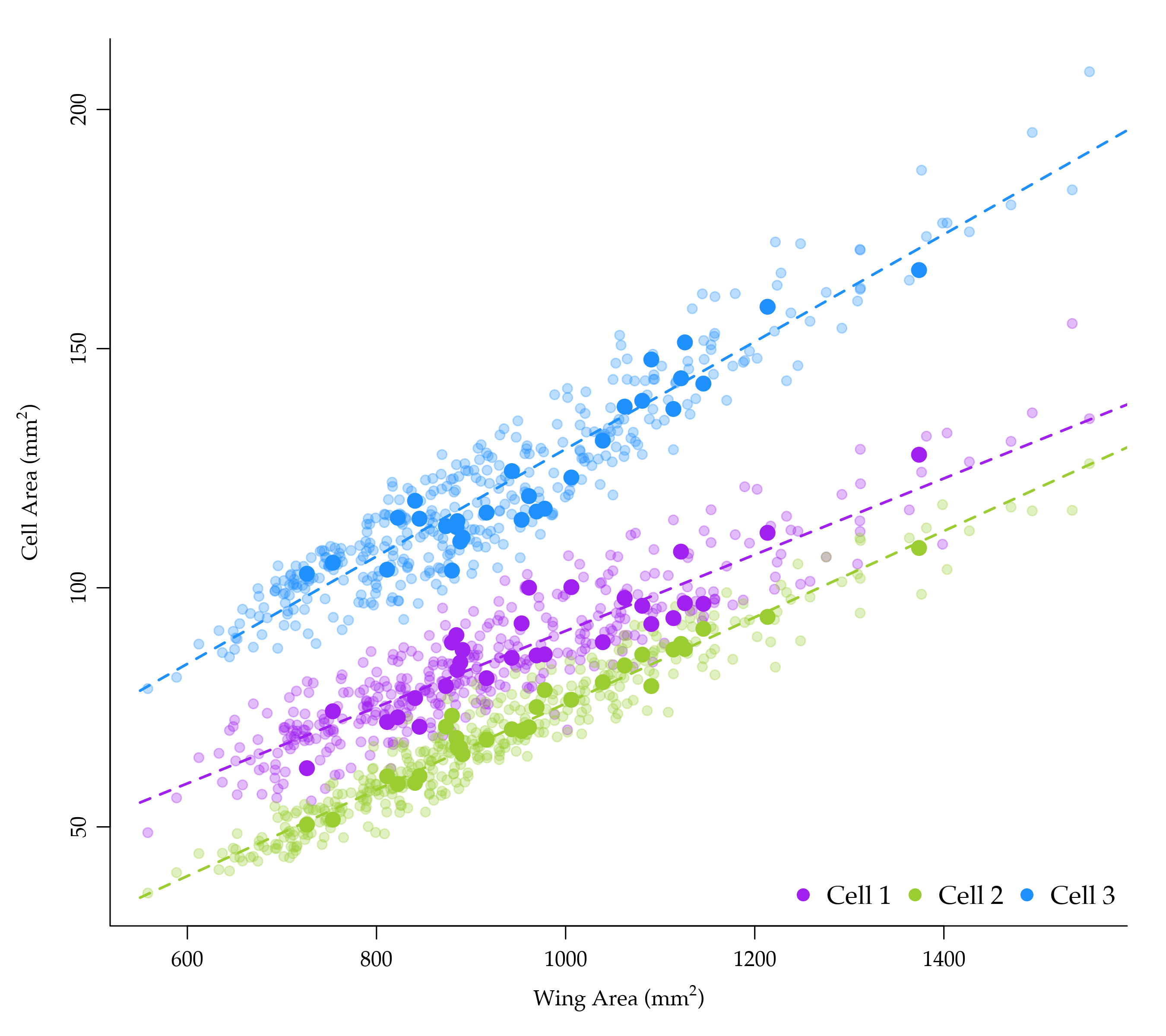
***

Figure S11. Correlation between each of the cells and wing area.


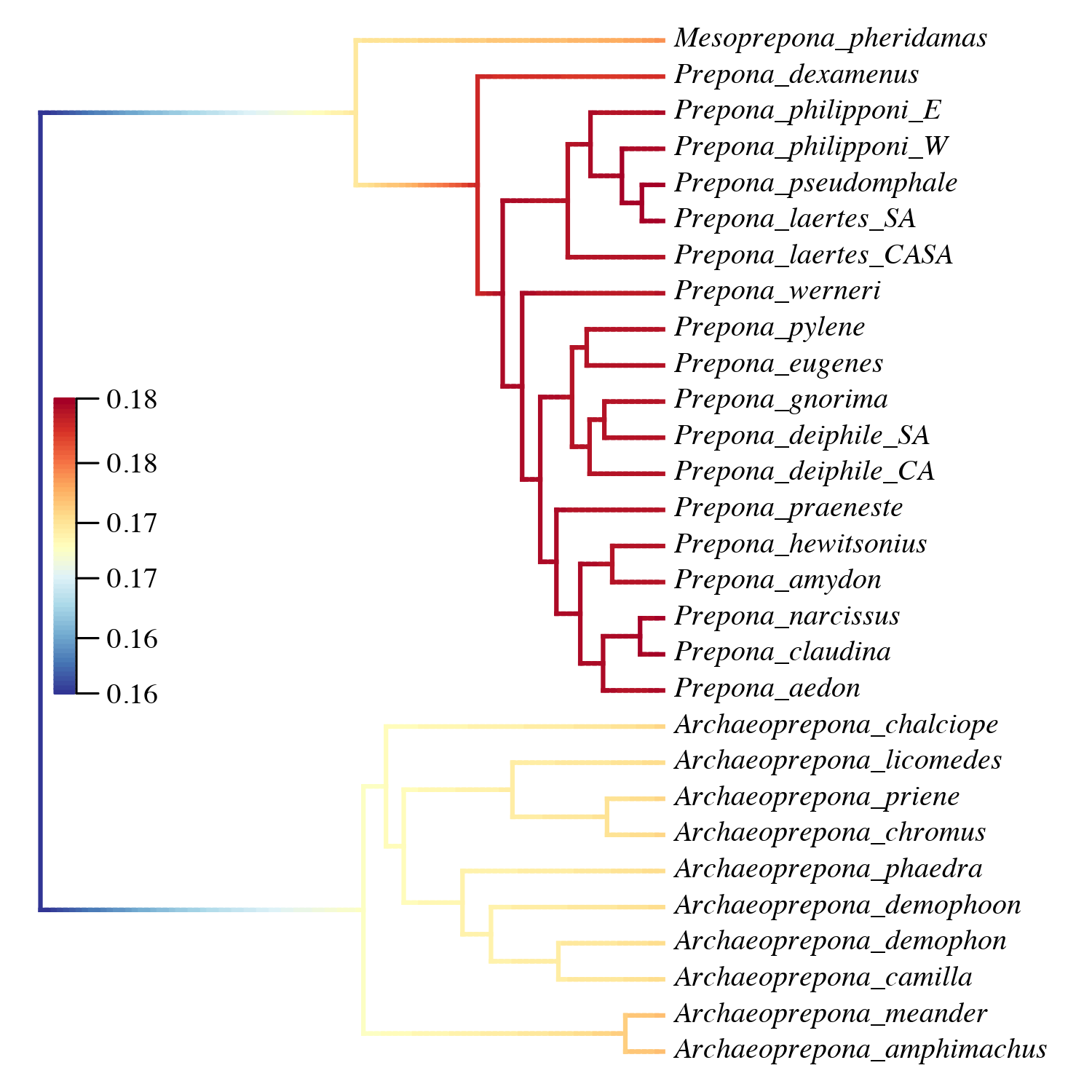


Figure S12. Diversification rates estimated by BAMM.
