# Supplementary material for: The roles of wing color pattern and geography in the evolution of Neotropical Preponini butterflies
Source: Ecol Evol. 2020 Oct 3;10(23):12801–16. doi: 10.1002/ece3.6816 (PMC7713932; doi:10.1002/ece3.6816)
Supplement: Supplementary file 3 — AppendixS1 [file ECE3-10-12801-s003.docx]

**Appendix S1**

**Methods**

***Diversification dynamics estimation.*** To test if there has been a change in diversification rates through the evolutionary history of Preponini butterflies, we used the CRT by calculating the gamma statistic (Pybus & Harvey, 2000). The CRT evaluates if the positions of the internal nodes are closer or further from the root than expected under a pure birth process. We then estimated speciation rate assuming a pure birth process and given the clade age and species richness using the estimator of Magallón and Sanderson (2001).

We also used BAMM 2.5.0 to identify rate shifts across the Preponini phylogeny (Rabosky, 2014). BAMM finds the shifts on the diversification process using a reversible jump Markov Chain Monte Carlo process to estimate a posterior distribution of the number and location of the rate changes. We used BAMMtools 2.2 (Rabosky et al., 2014) to analyze the output and obtain appropriate priors for the estimation (Mitchell & Rabosky, 2017). We ran four chains of 220 million generations each sampled every 10.000 steps and a burn-in of 10% and used ‘coda’ (Plummer et al., 2006) to asses convergence of the chains and check the effective sample size.

We used MEDUSA as an alternative to BAMM to estimate shifts in diversification rates across the tree. The approach adds a series of diversification breakpoints which estimates the parameters under a Yule, a birth-death process or mixed models. It then returns the best combination of diversification breakpoints by comparing the model with increased number of parameters to the simpler constant diversification model (Alfaro et al., 2009).

Finally, we fitted a 3 million year gridded hierarchical birth-death model to estimate pulled diversification rates which avoids identifiability problems between speciation and extinction estimates using the package ‘castor’ for R (Louca & Doebeli, 2017).

***Wing color pattern evolution.*** All species were represented by at least six individuals except for *Prepona philipponi W* and *Archaeoprepona priene*, for which only one and two specimens respectively were available. Even though species in the tribe are not considered to be sexually dimorphic, we selected only male specimens for analyses because of their better representation in collections and to avoid the possibility of slight variations in color between sexes.

The collection date of specimens photographed ranged between 1910 to 2017, with almost 70% of the specimens collected after 1980. The collection date varied among species but we attempted to maintain an approximately similar distribution of collection dates so that error due to potential reduction in color intensity was similar across the tips of the tree (Figure S1).

Since the wing veins which delineate the three regions are homologous across species, the same region in the wing was measured consistently, irrespective of wing size or shape. Similarly, since wing cell area scales with wing size, a similar proportion of the wing area was measured for all specimens. The correlation between the area of each of the cells and total wing area was larger than 0.906 for every cell and represents on average 10% of the total wing area (Figure S11; Cell 1 – R = 0.906, Cell 1 Area = 11.2 + 0.08*Wing Area; Cell 2 – R = 0.96, Cell 2 Area = -14.4 + 0.09*Wing Area; Cell 3 – R = 0.94, Cell 3 Area = 16.7 + 0.11*Wing Area) (Figure S11).

**Results**

***Diversification dynamics estimation.*** According to the gamma statistic, diversification rates have been constant through the evolutionary history of Preponini (γ = -0.18, p = 0.85). Magallón and Sanderson’s estimator (2001) suggested that diversification rate is 0.1 LogSpecies/Ma. Similarly, BAMM found no change in diversification with a mean rate of 0.17 LogSpecies/Ma (Credible Interval 0.11 – 0.26; Table S16, Figure S12). In contrast, both the LTT and MEDUSA suggested an increase in diversification rates (Figure 2). MEDUSA showed that diversification increased from 0.08 (95% CI: 0.04 - 0.14) to 0.25 (95% CI: 0.14 – 0.39) at 7.05 Ma, which corresponds to the clade sister to *Prepona dexamenus* (ΔAIC = 4.23, lnLik one rate = -81.39, lnLik two rates = 77.16; Figure 2). Pulled diversification rate estimates also suggest constant speciation and extinction rates through time with pulled speciation rate estimate of 0.14 LogSpecies/Ma and pulled extinction rate estimate of 0.05 LogSpecies/Ma. We highlight that speciation rates estimated by all models are around the same magnitude (0.12-0.17 LogSpecies/Ma)

**Discussion**

***Diversification dynamics estimation***. CR and BAMM’s results suggest constant rates of evolution in Preponini. In contrast, we observe a deviation in the LTT plot supported by MEDUSA which shows an increase in diversification rate ~7 Ma (middle Miocene to late Miocene-Pliocene) in the clade with drastic coloration change (i.e. clade sister to *Prepona dexamenus*), supporting other butterfly studies in which diversification rates are influenced by phenotype and/or biogeography (e.g. Chazot et al., 2014; Jiggins et al., 2006). Support for our hypothesis would be provided by an increase in phenotypic evolutionary rate followed by the increase in diversification. Contrastingly, our MEDUSA analyses suggest an earlier increase in diversification rate followed by a later change in phenotypic rate (Figure 2). The trait-dependent diversification analyses (Table 4) supported the later result since we did not find any correlation between diversification rate and the color traits measured. Thus, for color pattern traits, other explanations are required to better understand the drastic shift in coloration observed. Alternatively, we found congruency between the uplift of the Andes and closure of the Panama Isthmus and the slight change in diversification rate at ~ 7 Ma.

We carefully discuss these methods since Kodandaramaiah and Murali (2018) show that constant diversification rates reported by both BAMM and MEDUSA may be misleading, encourage further exploration. Estimation of diversification rates is widely used in the scientific literature since it provides key insight into how traits or environmental factors have shaped the evolutionary trajectories of many groups (Morlon, 2014; Ricklefs, 2007). Many methods attempt to understand the speciation process, and although some perform better than others for specific cases, none has lacked criticism, and BAMM and MEDUSA are not the exception (May & Moore, 2016; Meyer et al., 2018). Still, both approaches are currently used for phylogenies of all sizes, across taxonomic levels and in a wide diversity of groups. We acknowledge the contrasting signal obtained by these methods and recognize that having a small phylogeny might add to the uncertainty of the results. We are cautious with our results and aware of the shortcomings of these methods, and thus we use this conclusion as hypothesis generator that can be tested with novel methods in future studies.

**References**

Alfaro, M. E., Santini, F., Brock, C., Alamillo, H., Dornburg, A., Rabosky, D. L., Carnevale, G., & Harmon, L. J. (2009). Nine exceptional radiations plus high turnover explain species diversity in jawed vertebrates. *Proceedings of the National Academy of Sciences of the United States of America*, *106*(32), 13410–13414. https://doi.org/10.1073/pnas.0811087106

Chazot, N., Willmott, K. R., Endara, P. G. S., Toporov, A., Hill, R. I., Jiggins, C. D., & Elias, M. (2014). Mutualistic Mimicry and Filtering by Altitude Shape the Structure of Andean Butterfly Communities. *The American Naturalist*, *183*(1), 26–39. https://doi.org/10.1086/674100

Jiggins, C. D., Mallarino, R., Willmott, K. R., & Bermingham, E. (2006). The phylogenetic pattern of speciation and wing pattern change in neotropical *Ithomia* butterflies (Lepidoptera: nymphalidae). *Evolution*, *60*(7), 1454–1466.

Kodandaramaiah, U., & Murali, G. (2018). What affects power to estimate speciation rate shifts? *PeerJ*, *6:e5495*. https://doi.org/10.7717/peerj.5495

Louca, S., & Doebeli, M. (2017). Efficient comparative phylogenetics on large trees. *Bioinformatics*, *34*(6), 1053–1055. https://doi.org/10.1093/bioinformatics/btx701

Magallon, S., & Sanderson, M. J. (2001). Absolute diversification rates in angiosperm clades. *Evolution*, *55*(9), 1762–1780. https://doi.org/10.1111/j.0014-3820.2001.tb00826.x

May, M. R., & Moore, B. R. (2016). How Well Can We Detect Lineage-Specific Diversification-Rate Shifts? A Simulation Study of Sequential AIC Methods. *Systematic Biology*, *65*(6), 1076–1084. https://doi.org/10.1093/sysbio/syw026

Meyer, A. L. S., Román‐Palacios, C., & Wiens, J. J. (2018). BAMM gives misleading rate estimates in simulated and empirical datasets. *Evolution*, *72*(10), 2257–2266. https://doi.org/10.1111/evo.13574

Mitchell, J. S., & Rabosky, D. L. (2017). Bayesian model selection with BAMM: effects of the model prior on the inferred number of diversification shifts. *Methods in Ecology and Evolution*, *8*(1), 37–46. https://doi.org/10.1111/2041-210X.12626

Morlon, H. (2014). Phylogenetic approaches for studying diversification. *Ecology Letters*, *17*(4), 508–525. https://doi.org/10.1111/ele.12251

Plummer, M., Best, N., Cowles, K., & Vines, K. (2006). CODA: convergence diagnosis and output analysis for MCMC. *R News*, *6*(1), 7–11.

Pybus, O. G., & Harvey, P. H. (2000). Testing Macro-Evolutionary Models Using Incomplete Molecular Phylogenies. *Proceedings: Biological Sciences*, *267*(1459), 2267–2272. JSTOR.

Rabosky, D. L. (2014). Automatic Detection of Key Innovations, Rate Shifts, and Diversity-Dependence on Phylogenetic Trees. *PLoS ONE*, *9*(2). https://doi.org/10.1371/journal.pone.0089543

Rabosky, D. L., Grundler, M., Anderson, C., Title, P., Shi, J. J., Brown, J. W., Huang, H., & Larson, J. G. (2014). BAMMtools: an R package for the analysis of evolutionary dynamics on phylogenetic trees. *Methods in Ecology and Evolution*, *5*(7), 701–707. https://doi.org/10.1111/2041-210X.12199

Ricklefs, R. E. (2007). Estimating diversification rates from phylogenetic information. *Trends in Ecology & Evolution*, *22*(11), 601–610. https://doi.org/10.1016/j.tree.2007.06.013
